# Potent and broad neutralization of SARS-CoV-2 variants of concern (VOCs) including omicron sub-lineages BA.1 and BA.2 by biparatopic human VH domains

**DOI:** 10.1016/j.isci.2022.104798

**Published:** 2022-07-20

**Authors:** Chuan Chen, James W. Saville, Michelle M. Marti, Alexandra Schäfer, Mary Hongying Cheng, Dhiraj Mannar, Xing Zhu, Alison M. Berezuk, Anupam Banerjee, Michele D. Sobolewski, Andrew Kim, Benjamin R. Treat, Priscila Mayrelle Da Silva Castanha, Nathan Enick, Kevin D. McCormick, Xianglei Liu, Cynthia Adams, Margaret Grace Hines, Zehua Sun, Weizao Chen, Jana L. Jacobs, Simon M. Barratt-Boyes, John W. Mellors, Ralph S. Baric, Ivet Bahar, Dimiter S. Dimitrov, Sriram Subramaniam, David R. Martinez, Wei Li

**Affiliations:** 1 Center for Antibody Therapeutics, Division of Infectious Diseases, Department of Medicine, University of Pittsburgh Medical School, Pittsburgh, PA, USA; 2 Department of Biochemistry and Molecular Biology, University of British Columbia, Vancouver, BC V6T 1Z3, Canada; 3 Department of Infectious Diseases and Microbiology, School of Public Health, University of Pittsburgh, Pittsburgh, PA, USA; 4 Department of Epidemiology, University of North Carolina at Chapel Hill, Chapel Hill, NC 27599, USA; 5 Department of Computational and Systems Biology, School of Medicine, University of Pittsburgh, Pittsburgh, PA, USA; 6 Division of Infectious Diseases, Department of Medicine, University of Pittsburgh School of Medicine, Pittsburgh, PA, USA; 7 Abound Bio, Pittsburgh, PA, USA; 8 Gandeeva Therapeutics, Inc., Vancouver, BC, Canada

**Keywords:** Immunology, Virology

## Abstract

The emergence of SARS-CoV-2 variants of concern (VOCs) requires the development of next-generation biologics with high neutralization breadth. Here, we characterized a human V_H_ domain, F6, which we generated by sequentially panning large phage-displayed V_H_ libraries against receptor binding domains (RBDs) containing VOC mutations. Cryo-EM analyses reveal that F6 has a unique binding mode that spans a broad surface of the RBD and involves the antibody framework region. Attachment of an Fc region to a fusion of F6 and ab8, a previously characterized V_H_ domain, resulted in a construct (F6-ab8-Fc) that broadly and potently neutralized VOCs including Omicron. Additionally, prophylactic treatment using F6-ab8-Fc reduced live Beta (B.1.351) variant viral titers in the lungs of a mouse model. Our results provide a new potential therapeutic against SARS-CoV-2 variants including Omicron and highlight a vulnerable epitope within the spike that may be exploited to achieve broad protection against circulating variants.

## Highlights

• Identification of a human V_H_ with broad neutralization against SARS-CoV-2 VOCs
• CryoEM reveals a unique binding paratope of F6 involving the framework region
• The biparatopic antibody (F6-ab8-Fc) enhances the neutralization potency
• F6-ab8-Fc reduces disease burden and protects mice from the Beta variant mortality

## Graphical abstract

**Figure:**
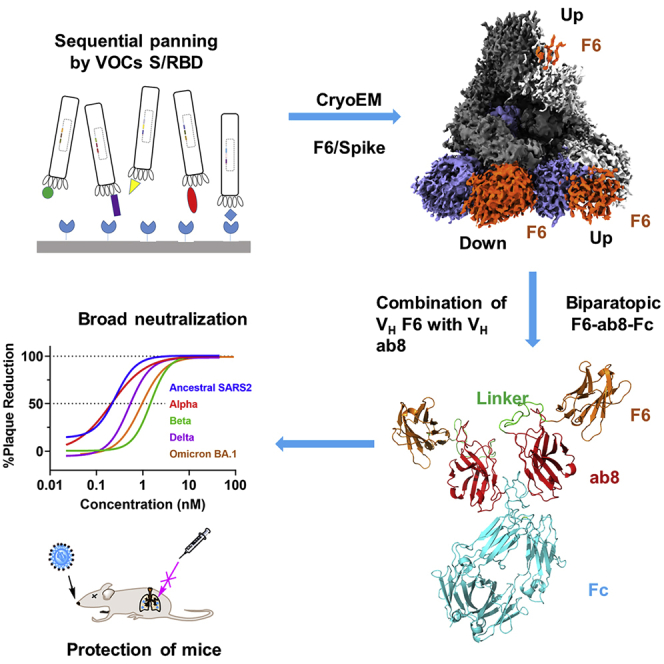

## Introduction

Since the start of the coronavirus disease 2019 (COVID-19) pandemic (Cui et al., 2019; Dong et al., 2020, 2021; Zhu et al., 2020), more than 532 million cases and 6.3 million deaths have been confirmed as of May 24^th^, 2022. To treat infections by severe acute respiratory syndrome coronavirus 2 (SARS-CoV-2), the causative agent of COVID-19, various therapeutics have been explored, such as convalescent patient sera (Klassen et al., 2021), neutralizing antibodies (nAbs) (Bracken et al., 2021; Cao et al., 2020b; Hansen et al., 2020; Liu et al., 2020a; Lv et al., 2020; Noy-Porat et al., 2020; Pinto et al., 2020; Robbiani et al., 2020; Schoof et al., 2020; Yuan et al., 2020), and small antiviral molecules (Cao et al., 2020a; Chan et al., 2020; Glasgow et al., 2020; Grein et al., 2020; Miao et al., 2020; Monteil et al., 2020). The spike glycoprotein (S protein), which engages the human ACE 2 (hACE2) receptor (Kuba et al., 2005), is a major target for Ab-mediated neutralization. nAbs that block SARS-CoV-2 spike protein from binding or mediating membrane fusion to ACE2 and are promising therapeutic candidates. Several nAbs have received emergency use authorization (EUA) in the United States (Dong et al., 2021; Kim et al., 2021; Schoof et al., 2020).

The receptor binding domain (RBD) within the subunit 1 (S1) region of the spike protein exhibits a high degree of mutational plasticity and is prone to accumulate mutations that lead to partial or full immune escape (Andreano et al., 2021; Geers et al., 2021; Lazarevic et al., 2021; Prevost and Finzi, 2021; Van Egeren et al., 2021; Weisblum et al., 2020; Zhou et al., 2021). The WHO (WHO) has designated several SARS-CoV-2 lineages as Variants of Concern (VOCs), which are more transmissible, more pathogenic, and/or can partially evade host immunity, including the Alpha, Beta, Gamma, Delta variants, and the recently identified Omicron variant (Baum et al., 2020; Ho et al., 2021; Jiang et al., 2021; Wang et al., 2021; Wibmer et al., 2021; Zhou et al., 2021). Some pan-sarbecovirus mAbs have been demonstrated to retain their neutralization activity against these VOCs (Martinez et al., 2022). The Omicron variant (BA.1) is heavily mutated compared to the ancestral lineage (Wuhan-Hu-1) and contains 30 amino acid substitutions in the spike protein, with 15 mutations localizing to the RBD (Callaway, 2021). Some of these mutations have been predicted or demonstrated to either enhance transmissibility (Grabowski et al., 2022) or to contribute to escape from many nAbs that were raised against the original (Wuhan-Hu-1) or early VOCs lineages of SARS-CoV-2. Recently, Omicron has further evolved into several sub-lineages including BA.2-BA.5, which demonstrate higher transmission and enhanced pathogenicity relative to BA.1 (Kumar et al., 2022). Compared to BA.1, the BA.2 RBD contains three more mutations (T376A, D405N, and R408S), but lacks the BA.1-specific G446S and G496S mutations. Based on the parental BA.2 lineage, the new sub-lineages BA.2.12.1, BA.2.13, BA.4, and BA.5 harbor the L452Q, L452M, and L452R + F486V RBD mutations, respectively. The different mutations in the spike RBD of the new omicron sub-lineages may impart a distinct escape from humoral immunity (Cao et al., 2022). The continuous evolution and emergence of VOCs that can partially evade host immunity require the development of Abs with broad neutralizing activity that can block or reduce disease burden. Additionally, multi-specific Abs or Ab cocktails hold promise to resist mutational escape by targeting multiple epitopes on the SARS-CoV-2 spike protein (Baum et al., 2020; Hansen et al., 2020). Several bispecific Abs have broad neutralization activity against SARS-CoV-2 variants (Bracken et al., 2021; Cho et al., 2021; De Gasparo et al., 2021); therefore, the generation of bispecific or multi-specific nAbs to target variants that otherwise evade immune response is a viable therapeutic strategy.

In this study, we identify a V_H_ domain (V_H_ F6) that shows broad neutralizing activity against SARS-CoV-2 variants including Alpha, Beta, Gamma, Delta, and Omicron BA.1 and BA.2 VOCs. V_H_ F6 binds a relatively conserved portion of the receptor binding motif (RBM), using a unique framework region (FR)-driven paratope. By combining V_H_ F6 with our previously identified Ab, V_H_ ab8, we developed a biparatopic Ab (F6-ab8-Fc), which exhibits potent neutralizing activity against all tested SARS-CoV-2 variants including the Omicron BA.1 and BA.2 VOCs. Prophylactic dosing with F6-ab8-Fc reduced viral titers in the lungs of a mouse model and high therapeutic doses of F6-ab8-Fc protected against mortality. Our study identifies a novel broadly neutralizing V_H_ domain Ab with a unique paratope and provides a potent biparatopic Ab (F6-ab8-Fc) against all tested SARS-CoV-2 variants, including the presently dominant Omicron BA.1 and BA.2 sub-lineages.

## Results

### Identification of a novel antibody domain (V_H_ F6) which binds to most prevalent receptor binding domain mutants and neutralizes SARS-CoV-2 variants including omicron BA.1 and BA.2

**Figure 1:**
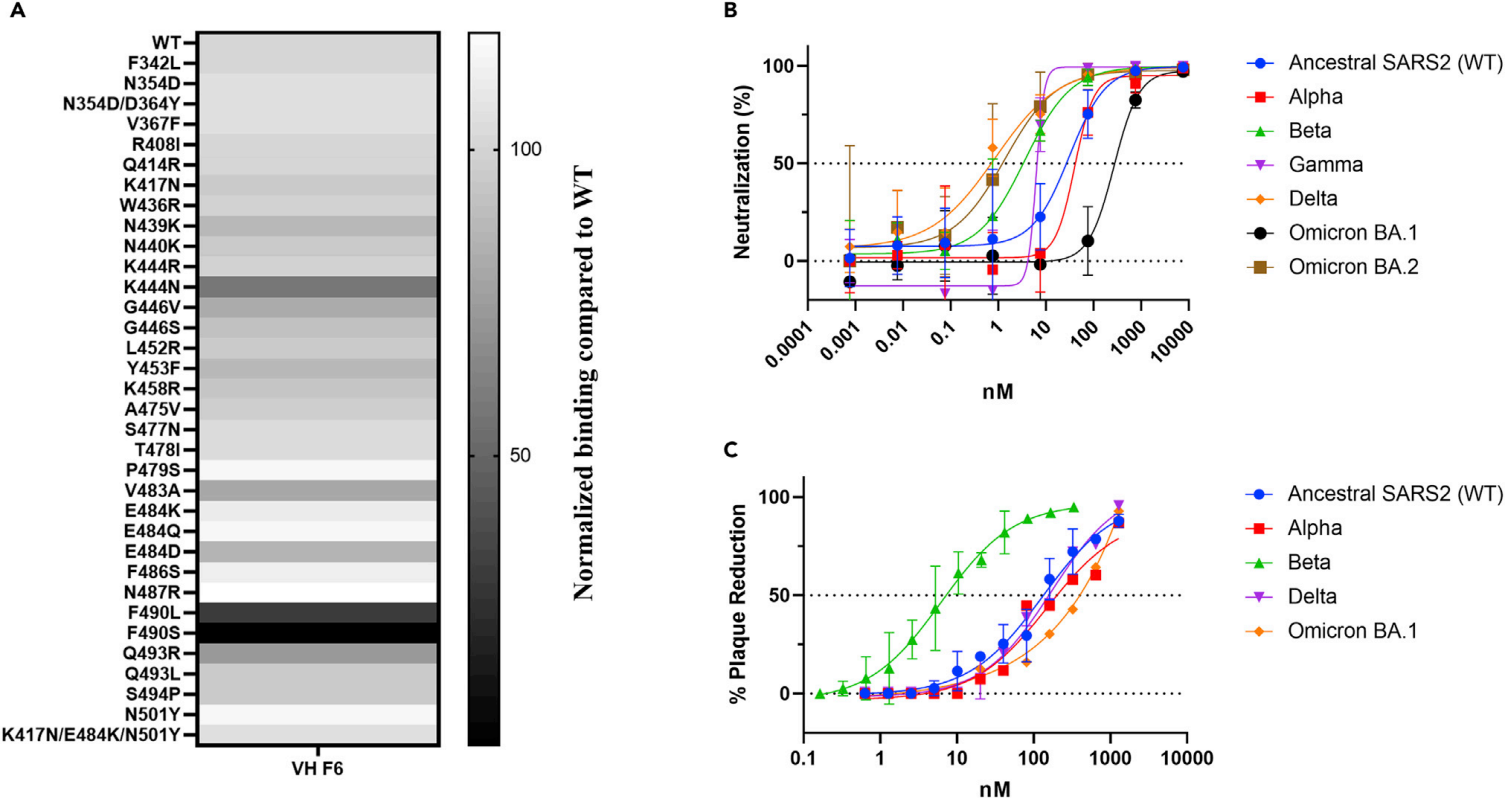
V_H_ F6 binds to prevalent RBD mutants and neutralizes SARS-CoV-2 VOCs including Omicron BA.1 and BA.2 (A) Heatmap of V_H_ F6 binding to circulating RBD mutants. The binding of V_H_ F6 to RBD mutants was detected by ELISA and normalized by comparing area under the curves (AUCs) between mutant and wild-type RBD. (B) Neutralization of SARS-CoV-2 WT, Alpha, Beta, Gamma, Delta, and Omicron BA.1 and BA.2 variants pseudovirus neutralization assays by V_H_ F6. Experiments were repeated at least twice with triplicate and error bars denote ±SD, n = 3. (C) Neutralization of SARS-CoV-2 WT, Alpha, Beta, Delta, and Omicron BA.1 variants live virus by V_H_ F6. Experiments were repeated twice with triplicate and error bars denote ±SD, n = 3.

To identify cross-reactive V_H_ domains against SARS-CoV-2 VOCs, we adopted a sequential panning strategy to pan our in-house large V_H_ phage library. We used RBD containing the E484K mutation for the first round of panning, wild-type (WT) RBD for the second, and the spike protein S1 domain-containing K417N, E484K, and N501Y mutations for the third (Figure S1A). Following these three rounds of panning, a dominant clone, V_H_ F6, was identified by ELISA screening. V_H_ F6 bound to the WT and Beta RBDs with half-maximal binding concentrations (EC_50_) of 5.1 and 7.2 nM, respectively (Figure S1B). V_H_ F6 also bound to the WT, Alpha, and Beta S1 proteins (Figure S1C). To assess the cross-reactivity of V_H_ F6, we performed ELISA and pseudovirus and live-virus neutralization assays. V_H_ F6 bound to trimeric spike proteins from multiple SARS-CoV-2 VOCs including Alpha, Beta, Gamma, Kappa, and Delta variants (Figure S1D). Furthermore, we evaluated the ability of V_H_ F6 to bind RBDs containing single-point mutations at mutational sites commonly observed in currently circulating variants. V_H_ F6 bound to 35 out of the 37 assayed RBD mutations, with only F490S and F490L mutants escaping binding (Figures 1A and S1E). V_H_ F6 was able to neutralize ancestral SARS-CoV-2 (WT), Alpha, Beta, Gamma, and Delta spike pseudotyped viruses with a 50% inhibition concentration (IC_50_) of 31.08, 40.32, 3.62, 6.23, and 0.86 nM, respectively (Figure 1B). Furthermore, V_H_ F6 neutralized replication-competent SARS-CoV-2 live viruses, with IC_50_s of 129.8, 149, 6.18, and 169.9 nM for the parental Wuhan-1, Alpha, Beta, and Delta variants, respectively (Figure 1C). V_H_ F6 neutralized the Beta variant live virus more potently than other variants.

The Omicron variant escapes most mAbs that are in clinical use (Cameroni et al., 2022). V_H_ F6 bound the Omicron BA.1 RBD with an EC_50_ of 68.6 nM as tested by ELISA, which is consistent with the binding dissociation constant (*K*_*D*_ = 19.8 nM) obtained by BLItz (Figures S1F and 1G). Importantly, V_H_ F6 neutralized BA.1 pseudovirus with an IC_50_ of 268.9 nM (Figure 1B). V_H_ F6 neutralized BA.2 more potently than BA.1, with an IC_50_ of 1.38 nM (Figure 1B).

### Cryo-EM structure of the V_H_ F6 - beta variant spike protein complex reveals a unique FR-driven binding mode

**Figure 2:**
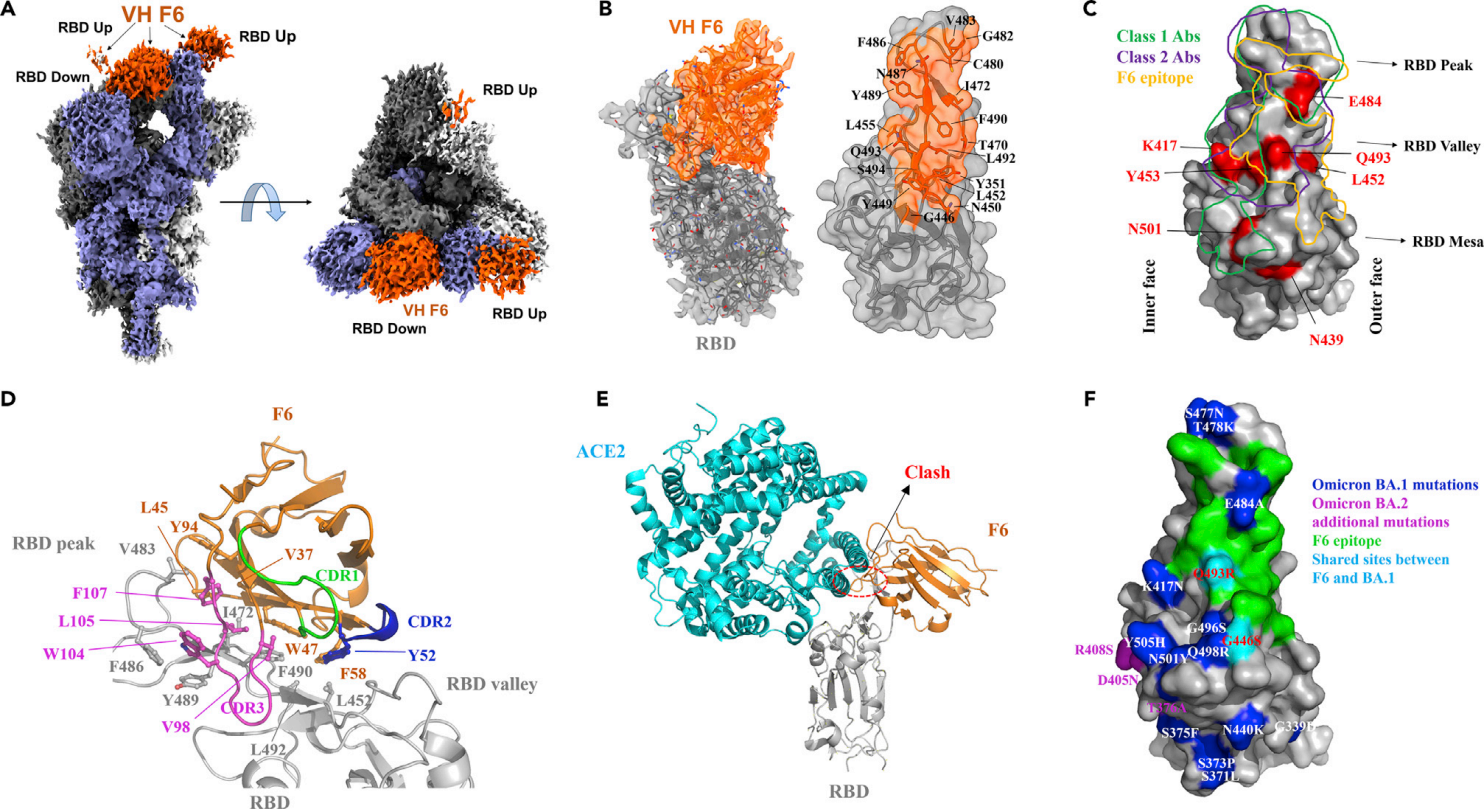
CryoEM structure of V_H_ F6 in complex with the SARS-CoV-2 Beta variant spike protein (A) Global cryoEM map of the Beta variant spike protein in complex with V_H_ F6. Density corresponding to the Beta variant trimer is colored in shades of gray and violet while density corresponding to V_H_ F6 molecules is colored in orange. (B) Left: Focus refined density map of the Beta variant RBD - V_H_ F6 complex with the docked atomic model. Right: Molecular surface representation of the epitope of V_H_ F6 on the Beta variant RBD. The side chains of residues within the binding footprint of V_H_ F6 are displayed and colored orange. (C) Footprints (i.e. surface binding areas/regions) of class 1 Abs (green), class 2 Abs (purple), and V_H_ F6 (orange) on the molecular surface of the SARS-CoV-2 RBD. Commonly mutated and antibody-evading mutations are colored in red. (D) Focused view of the atomic model at the V_H_ F6 - RBD interface. The side chains of discussed residues are shown, with the scaffold colored in orange, CDR1 green, CDR2 blue, CDR3 magenta, and the RBD gray. (E) Superposition of V_H_ F6-RBD (orange) and ACE2-RBD (cyan) complex atomic models. The RBD is shown in gray and the ACE2-RBD model was derived from PBD ID: 6m0j. (F) Mapping the Omicron BA.1 and BA.2 mutations onto the RBD structure with comparison to the F6 epitope. The green surface region represents the F6 footprint/epitopes on RBD, while the blue spots stand for the BA.1 mutations. The additional BA.2 mutations T376A, D405N, and R408S mutational sites are colored by the magenta.

To gain insights into the broad neutralization exhibited by V_H_ F6, we solved the cryo-electron microscopy (cryoEM) structure of V_H_ F6 bound to a prefusion stabilized Beta spike trimer at a global resolution of 2.8 Å (Figure S2 and Table S1). The Beta variant trimer was chosen for structural analysis as it contains K417N, E484K, and N501Y mutations, different combinations of which are present in other variants (Alpha, Gamma, and Omicron). CryoEM reconstruction revealed density for three bound V_H_ F6 molecules with strong density observed for V_H_ F6 binding to a “down” RBD, and moderate or weak densities for two V_H_ F6 molecules binding “up” RBDs (Figure 2A). The strong density for V_H_ F6 bound to the “down” RBD enabled focused refinement, providing a local resolution density map at 3.0 Å and enabling detailed analysis of the V_H_ F6 epitope (Figure 2B).

V_H_ F6 binding spans the RBD “peak” and “valley” regions, with its footprint skewed toward the RBD “outer face” (Figures 2B and 2C). This interface is exposed in both “up” and “down” RBD conformations, explaining how V_H_ F6 binds to both states simultaneously. Interestingly, the framework regions (FRs) of F6—a heavy-chain (V_H_) only Ab—expands the interaction interface beyond the conventional complementarity-determining regions (CDRs) (Figure 2D). Specifically, the hydrophobic FR2 residues present a hydrophobic core that associates with hydrophobic RBD residues which line the RBD peak and valley regions. This large FR engagement contributes to an interaction area that accounts for up to 36% of the total antibody paratope. Such substantial involvement of FRs causes V_H_ F6 to adopt an atypical perpendicular binding angle relative to the RBD, with its FR2, FR3, and CDR3 wrapping around the RBD peak (Figure 2D). In addition to FRs, CDR2 and CDR3 also contribute to the RBD binding interaction via hydrogen bonding, π-π stacking, and van der Waals interactions (Figures S3C–S3E). Owing to its positioning toward the RBD outer edge, the V_H_ F6 footprint only slightly overlaps with the hACE2 binding interface, potentially rationalizing its weaker RBD binding competition with hACE2 as compared to ab8 (Li et al., 2020) (Figures S3A, S3B and 2E).

The V_H_ F6-bound Beta spike protein structure rationalizes the broad activity of V_H_ F6 against various RBD mutants. Residues K417, N501, and E484—frequently mutated sites in VOCs and imparting escape from several nAbs—are not within the V_H_ F6 epitope (Figure 2C). The RBD residue Q493, which is mutated in the Omicron variant and induces escape from the clinical Ab REGN10933 (Starr et al., 2021; Zhu et al., 2021), is located within the V_H_ F6 epitope and forms hydrogen bonds with the main chain of G101 and S102 in the CDR3 (Figure S3C). Despite these specific hydrogen bonds, the Q493 R/L mutations did not significantly impact V_H_ F6 binding (Figure 1A), potentially reflecting either the plasticity or small overall contribution of this hydrogen bonding interaction. Residue L452—which is mutated to L452R in Delta and Kappa variants—is located within the periphery of the V_H_ F6 epitope and may contribute hydrophobic interactions with the V_H_ F6 residue F58 (Figure S3D). The peripheral nature of this interaction may explain the marginal sensitivity of V_H_ F6 binding to the L452R mutation (Figure 1A). In contrast, F490L and F490S mutations attenuate and completely abrogate V_H_ F6 binding, respectively (Figure 1A), as can be rationalized by the location of F490 within both the FR and CDR3 binding interfaces (Figure S3E). The lack of significant interactions with VOC mutated residues provides a structural basis for the broad activity of F6.

The resolved F6/Beta spike structure may also explain the binding and neutralization of V_H_ F6 to Omicron BA.1 and BA.2. According to the resolved F6/Beta RBD, 13 out of 15 omicron RBD mutations are located outside of the F6 epitope (Figure 2F), and the remaining two mutations, G446S and Q493R are in the peripheral region of the F6 footprint. Importantly, our RBD mutants ELISA showed the G446S and Q493R mutations did not significantly disturb F6 binding (Figure 1A). Structure modeling and molecular dynamics (MD) simulations were performed to examine the interfacial interactions and showed that the complex formed between the Omicron variant RBD and F6 stably retained the same structural features as the cryo-EM resolved F6-Beta RBD complex in triplicate runs of 800 ns. The mutation sites Q493R and Q498R intermittently formed new compensating salt bridges. Simulations and binding energy calculations repeated for the complexes of F6 with Beta and Omicron variants led to respective K_D_ values of 12.2 ± 3.1 nM and 15.5 ± 3.3 nM, which is in line with the BLItz K_D_ (Figure S4). The additional BA.2 RBD mutations (T376A, D405N, and R408S) are distal from the F6 epitope, likely rationalizing the cross-reactivity of V_H_ F6 against BA.2.

### Generation of a biparatopic antibody with enhanced neutralization of SARS-CoV-2 variants of concerns

**Figure 3:**
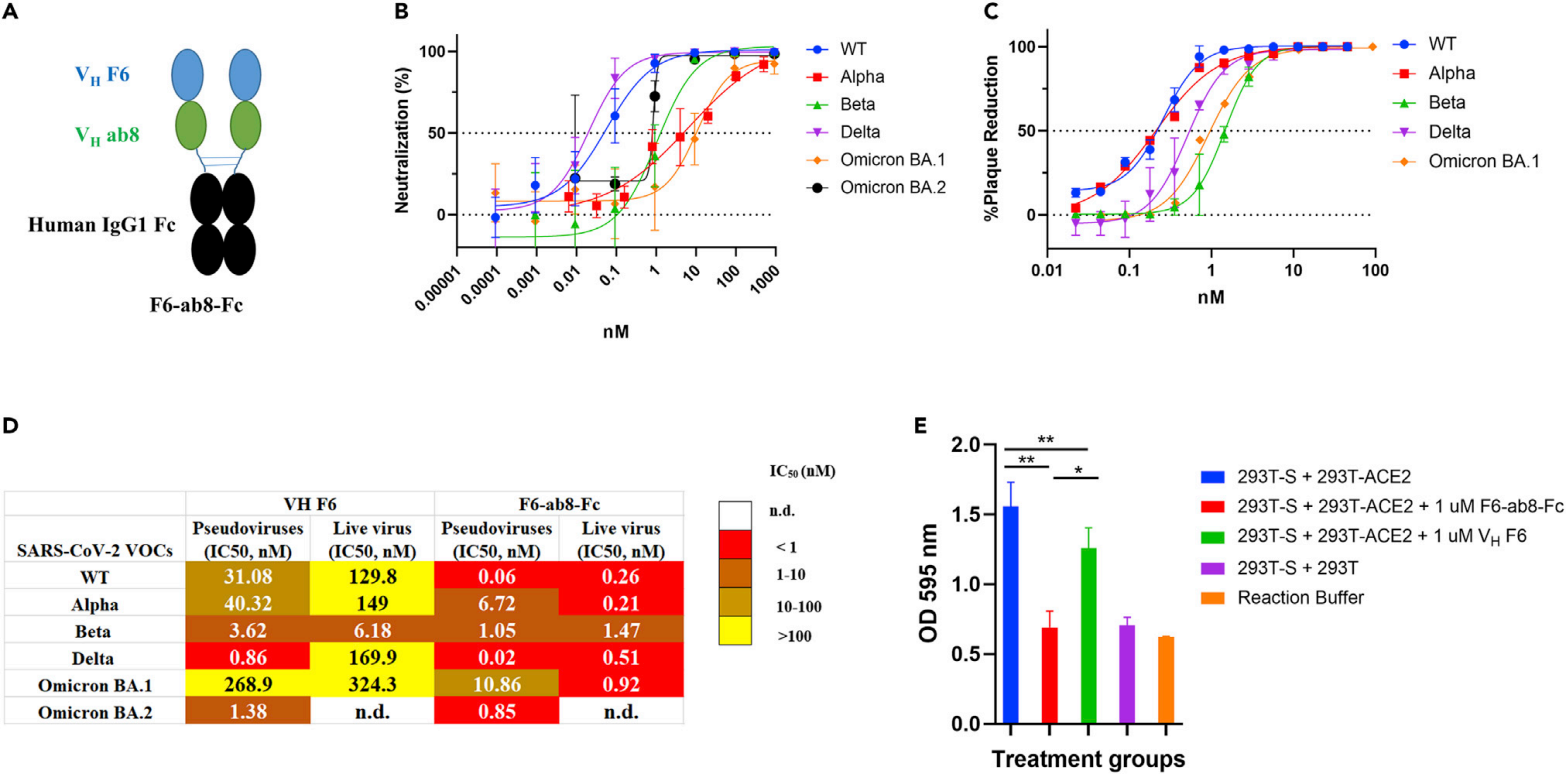
Construction of a biparatopic antibody (F6-ab8-Fc) that neutralizes various SARS-CoV-2 VOCs including Omicron BA.1 and BA.2 as measured by pseudovirus and live virus neutralization, and cell-cell fusion assays (A) The scheme of the biparatopic antibody F6-ab8-Fc containing a tandem VH (F6-ab8) at the N terminal of the human IgG1 Fc. (B-D) Neutralization of SARS-CoV-2 WT, Alpha, Beta, Delta and Omicron BA.1 and BA.2 variants pseudoviruses (B) and live viruses (C) by F6-ab8-Fc. Experiments were repeated at least twice in triplicate and error bars denote mean ±1 SD, n = 3. D. Comparisons of virus neutralization IC_50_s of V_H_ F6 and F6-ab8-Fc by both pseudovirus and live virus neutralization assays. (E) Inhibition of cell-cell fusion by F6-ab8-Fc as tested by a β-gal reporter gene assay, in which 293T-Spike cells infected with vaccinia virus expressing T7 polymerase were incubated with 293T-ACE2 cells infected with vaccinia virus encoding the T7 promotor-controlled β-galactosidase. The cell-to-cell fusion signal was monitored by the β-galactosidase activity. The incubation of 293T-spike with 293T-ACE2 cells without additions of Abs is the positive control, while incubation of 293T-spike with 293T (without expressing ACE2) was set as the negative controls. Experiments were performed in triplicate, and the data were presented as mean ±1 SD, n = 3. The paired *Student t* test was used to evaluate statistical differences. ∗p < 0.05, ∗∗p < 0.01.

To expand the V_H_ F6 epitope, with the aim of decreasing the potential of mutational escape, we designed a biparatopic Ab and added V_H_ ab8, which is a nAb with a distinct and partially overlapping epitope compared to that of F6 (Figures S5A and S5B). Although ab8 is escaped by the Beta, Gamma, and Omicron variants (Figure S5C), ab8 is not escaped by the F490S and F490L mutations that ablate V_H_ F6 binding (Figure S5D). The biparatopic Ab was constructed by linking V_H_ F6 to V_H_ ab8 via a 5×(GGGGS) polypeptide linker with the C terminal fused to the human IgG1 fragment crystallizable region (Fc) (Figure 3A). Addition of an Fc region extends antibody serum half-life and enables effector function of the immune system via Fc receptors (Li et al., 2020). The biparatopic Ab, F6-ab8-Fc, bound to SARS-CoV-2 variant spike trimer proteins (Figure S6A). Additionally, F6-ab8-Fc is potently bound to the Omicron BA.1 RBD and Omicron BA.1 and BA.2 spike proteins as measured by ELISA and BLItz (Figures S6B–S6F). F6-ab8-Fc had higher binding affinity to the BA.2 spike relative to the BA.1 spike. F6-ab8-Fc potently neutralized WT, Alpha, Beta, and Delta SARS-CoV-2 variants as measured by pseudovirus and live-virus assays (Figures 3B–3D). Importantly, F6-ab8-Fc neutralized Omicron BA.1 and BA.2 sub-lineages with IC_50_s of 10.86 and 0.85 nM, respectively (Figure 3D). The higher neutralization potency against BA.2 correlates with the higher binding affinity of F6-ab8-Fc to the BA.2 spike relative to BA.1. Although V_H_ F6 neutralized Omicron BA.1 live-virus with an IC_50_ of 324.3 nM, F6-ab8-Fc was more potent against Omicron BA.1 and neutralized live virus with an IC_50_ of 0.92 nM.

The neutralization activity of F6-ab8-Fc was more potent than V_H_ F6 (Figure 3D). To dissect the neutralization mechanism of F6-ab8-Fc, we designed a set of F6 constructs to compare their neutralization potency to V_H_ F6 and F6-ab8-Fc against Omicron BA.1. These constructs include F6-F6 (two V_H_ F6 connected by a tandem polypeptide linker 5×(GGGGS), F6-Fc (an F6 fusion with an Fc using the same linker as that in F6-ab8-Fc), F6-F6-Fc (a bivalent F6 connected in a tandem manner followed by fusion with an Fc to achieve tetravalency). F6-F6 neutralized with higher potency than F6, and F6-F6-Fc had the highest potency against BA.1 (Figure S5F), indicating that both avidity and the addition of the Fc region may contribute to the antiviral activity. We also found that F6-Fc was more potent than F6-F6, and while both constructs are bivalent, the bulkier Fc may cause increased steric occlusion of hACE2 binding thereby enhancing neutralization activity. Interestingly, while the BA.1 variant is resistant to ab8, F6-ab8-Fc exhibited slightly higher neutralization potency as compared to F6-Fc against BA.1. This enhanced inhibition may be rationalized by ab8 increasing the molecular size and contributing to the steric effect. Additionally, structural modeling (Figure S4D) suggests that ab8 may play a role to modulate the spatial orientation of V_H_ F6 to facilitate the potential inter-spike crosslinking, contributing to the enhanced neutralization of F6-ab8-Fc. It also should be noted that F6-F6-Fc exhibits higher neutralization potency than F6-ab8-Fc against Omicron BA.1, indicating that the avidity effect outperforms the ab8-mediated neutralization enhancement.

In addition, a β-gal reporter gene quantitative cell-to-cell fusion assay (Liu et al., 2020b) showed that F6-ab8-Fc inhibited the fusion of 293T-spike and 293T-hACE2 overexpressing cells, and was more potent than V_H_ F6 (Figure 3E). The exact mechanism of F6-ab8-Fc inhibition of cell-cell fusion is currently unclear but may relate to its blockade of hACE2, and/or potential interference with the conformational change in spike or inactivation of spike before engaging host cells. The capacity of F6-ab8-Fc to inhibit cell-to-cell fusion may constitute another neutralization mechanism that may play an important role in live virus neutralization, in which cell-to-cell viral spread possibly occurs during multi-round replication cycles but probably does not occur in the one-round virion infection in the pseudovirus neutralization assay. This may partially explain the overall high neutralization potency of F6-ab8-Fc against SARS-CoV-2 variant live viruses.

Taken together, the avidity, steric blocking of receptor engagement, inhibition of cell-cell fusion, and/or possible cross-linking of inter-spike may collectively contribute to the high neutralization potency of F6-ab8-Fc.

### F6-ab8-Fc prophylactically and therapeutically reduces disease burden and protects from SARS-CoV-2 beta variant mortality in mice

**Figure 4:**
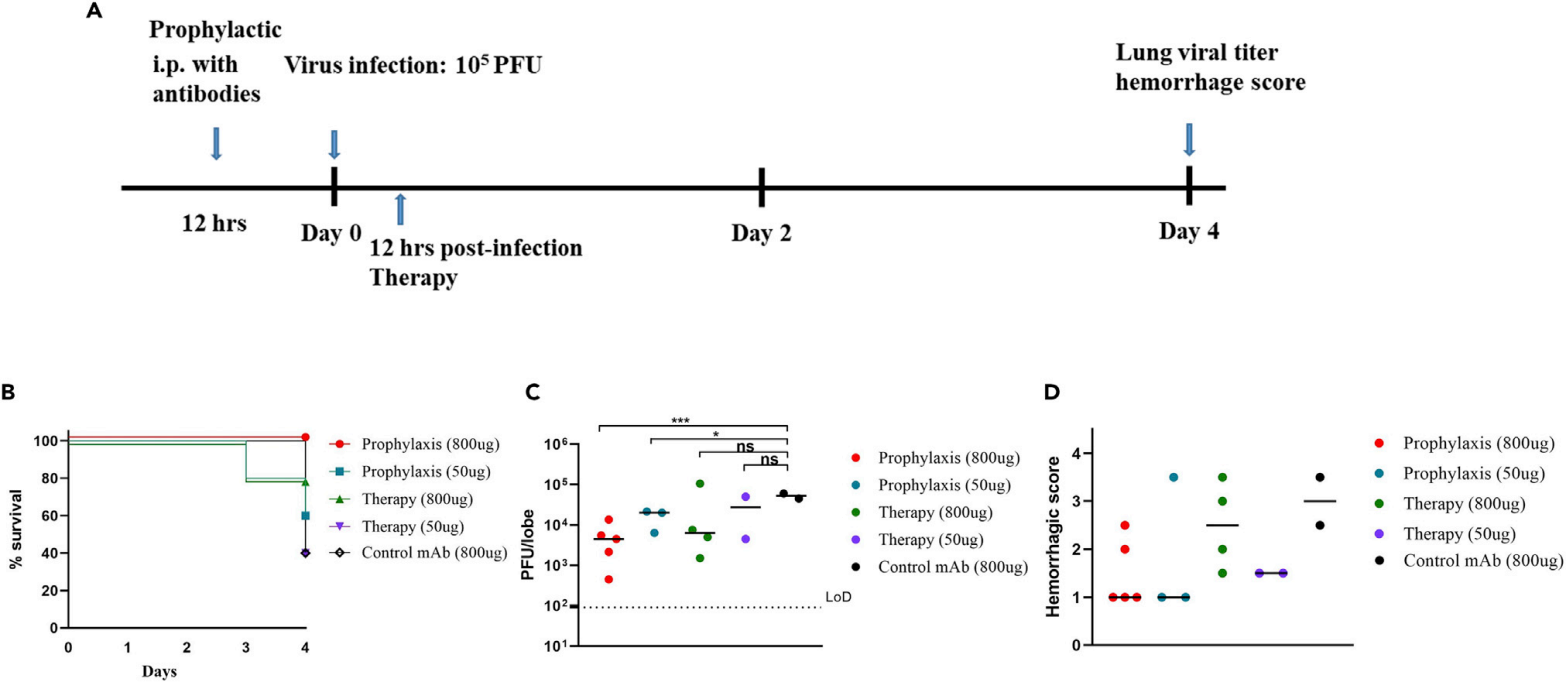
Evaluation of prophylactic and therapeutic efficacy of F6-b8-Fc in a mouse ACE2-adapted model (A) The overview of study design for evaluating F6-ab8-Fc efficacy in a SARS-CoV-2 mouse model. (B) Percent survival curves for each F6-ab8-Fc treatment group as indicated. (C) Lung viral titers (PFUs) in lung tissue for the F6-ab8-Fc treatment groups. The limit of detection (LoD) is 100 PFU/lobe. (D) Lung hemorrhage scores of live mice. *T* tests were used to evaluate statistical differences. ∗p < 0.05, ∗∗p < 0.01, ∗∗∗p < 0.001, ns. no significance.

To evaluate the prophylactic and therapeutic efficiency of F6-ab8-Fc *in vivo*, we used a mouse-adapted SARS-CoV-2 infection model (Martinez et al., 2021a, 2021b) The Beta variant was chosen for *in vivo* protection experiments because it is relatively difficult to neutralize (Collier et al., 2021; Wang et al., 2021). Groups containing five mice each were administered a high dose of 800 μg or a low dose of 50 μg F6-ab8-Fc 12 h pre- or 12 h post-SARS-CoV-2 mouse-adapted 10 (MA10) Beta variant challenge. Mice were monitored for signs of clinical disease and viral titers in the lungs were measured four days after infection (Figure 4A). Mice in the high-dose (800 μg) prophylaxis group were completely protected from mortality (0% mortality). In contrast, 20% mortality was observed in the 800 μg therapeutic group and 40% mortality was observed in the 50 μg prophylactic group. 60% mortality was observed in the 50 μg therapeutic and control mAb group (Figure 4B). Thus, F6-ab8-Fc can protect against mortality when given prophylactically at high doses. We observed more than one log reduction in viral titer in the high-dose prophylactic and therapeutic groups after four days (Figure 4C). Additionally, lung congestion scores, which is a gross pathologic score at the time of harvest, were lower in all four F6-ab8-Fc treated groups compared to the mAb control (Figure 4D). Our results indicate that F6-ab8-Fc reduces lung viral replication *in vivo,* with prophylactic treatment being more effective than therapeutic treatment.

## Discussion

The SARS-CoV-2 spike protein has accumulated numerous mutations that retain its ability to engage its receptor (hACE2), while evading neutralizing Abs (Mannar et al., 2022). The RBD is immunodominant and has accumulated several mutations that partially escape FDA-approved vaccines and the majority of mAbs in clinical use. A recent epitope binning and structural study classifies Ab epitopes across the RBD into six classes, with class 1-3 Abs targeting the top surface RBM region which competes with ACE2, and class 4/5 and class 6/7 Abs binding to the RBD outer and inner surfaces, respectively (Hastie et al., 2021). Class 1-3 Abs are most likely to be rendered ineffective by K417 N/T, E484K, and N501Y mutations which are found in Alpha, Beta, and Gamma variants. Currently, only a few RBM-targeting Abs are reported to neutralize the Omicron variant such as ACE2-mimicking Abs S2K146 (Park et al., 2022) and XGv347 (Wang et al., 2022).

In this study, we developed a novel single domain (human V_H_) Ab, F6 that can broadly neutralize Alpha, Beta, Gamma, Delta, and Omicron variants. V_H_ F6 targets a class-4 epitope that spans the RBD peak and valley outer-face, and partially overlaps with the hACE2 binding interface. Importantly, the CryoEM structure of V_H_ F6 in complex with the Beta spike protein revealed that VOC mutations lie either outside of the V_H_ F6 epitope (K417, E484, N501, N439) or within its periphery (L452, Q493, G446). The V_H_ F6 epitope bears a high degree of similarity to the full-length Ab A19-46.1, which can also neutralize the Omicron BA.1 variant (Zhou et al., 2022). Unlike A19-46.1, V_H_ F6 is not affected by the L452R mutation and can bind the RBD in both “up” and “down” conformations, probably owing to the lower steric hindrance associated with its small size. The ability of an antibody fragment to bind both “up” and “down” RBD states is an attractive property given that the accessibility of its epitope is independent of RBD conformation (Henderson et al., 2020). The resistance to L452R and F486S mutations (Figure 1A) may allow F6 to retain cross-reactivity to the newly emerging Omicron sub-lineages BA.4 and BA.5, which contain L452R/F486V mutations. Notably, V_H_ F6 adopts an uncommon angle of binding relative to the RBD, using its exposed FR regions and CDR3 to present a hydrophobic interaction interface. This interaction mode resembles that of llama/shark V_H_ Abs which use long CDR3s to fold against the FR2 region and collectively establish novel paratopes (Stanfield et al., 2007).

V_H_ F6 had increased neutralization activity against Beta and Delta pseudoviruses (Figures 1B and 3D), which may be explained by the higher binding affinity of V_H_ F6 to the Beta and Delta spike than to the ancestral spike (Figure S1D). The increased potency against the Beta variant may be attributed to spike mutations in Beta that increase V_H_ F6 binding, although this is currently unclear given that the Beta RBD mutations K417N/E484K/N501Y are not in the V_H_ F6 epitope. The higher binding of V_H_ F6 to the Delta spike may be explained by the L452R mutation, which is within the V_H_ F6 epitope, and R452 may impart new intermolecular interactions or enhance the electrostatic compatibility between V_H_ F6 and the Delta RBD. Intriguingly, V_H_ F6 neutralizes the Beta live virus more potently (IC_50_ = 6.18 nM) compared to other VOCs. The reasons for these differences in neutralization potency are unclear but could be related to the different spike mutations in VOCs which may influence spike conformation/processing on the virion surface. Interestingly, V_H_ F6 exhibits a higher neutralization potency for Omicron sub-lineage BA.2 than BA.1. The increased potency relative to BA.1 may be explained by the unique BA.1 mutation (G446S) within the F6 footprint that could disrupt F6-BA.1 binding.

V_H_ F6 primarily belongs to the class 4 Ab group, which also contains the highly potent and patient-derived Abs C002 (Barnes et al., 2020) and A19-46.1 (Zhou et al., 2022), and typically exhibits decreased binding to L452 and E484 mutated RBDs (Greaney et al., 2021). Additionally, the V_H_ F6 epitope partially overlaps with class 1-2 Abs which contain therapeutic Abs such as LY-CoV016 and REGN10933 (Greaney et al., 2021) (Figure 2C). The ability of the SARS-CoV-2 Omicron variant to escape class 1 and 2 Abs requires the development of Ab combinations (either cocktails or bior multi-specifics) targeting multiple epitopes. In this study, with the aim to target a broader epitope on the RBD, we generated a biparatopic Ab by combining F6 with the previously identified potent class 2 Ab domain V_H_ ab8 (Li et al., 2020). Although both Beta and Omicron variants were escaped by V_H_ Ab8, the biparatopic Ab, F6-ab8-Fc, potently neutralized all SARS-CoV-2 variants including Omicron BA.1 and BA.2. F6-ab8-Fc neutralized WT and Delta similarly, and neutralization of WT and Delta pseudoviruses was more potent than against other variants (Alpha, Beta, and Omicron BA.1) (Figure 3B), which may be ascribed to the synergy between F6 and ab8, as both F6 and ab8 potently neutralize WT and Delta, while ab8 is less potent against Alpha, and is completely escaped by Beta and Omicron (Figure S5C). However, higher neutralization as measured in pseudovirus neutralization assays did not always correlate with higher live virus neutralization (such as V_H_ F6 against Delta, and F6-ab8-Fc against WT and Delta in Figure 3D). These neutralization potency differences can be affected by various factors. One important factor is the spike distribution, density, pre- or post-fusion conformation, and the accessibility of neutralizing epitopes in the spike on the surface of virions. These variations can be affected by the different spike mutations in different SARS-CoV-2 VOCs. In addition, the different target cells used in the pseudovirus (293T-ACE2) and live virus (Vero E6) assays have different expression levels of hACE2 and cleavage proteases, which can also impact the neutralization potency. Another factor may be that the virus dose is dynamic during multiple replication cycles in live-virus neutralization assays, whereas the pseudovirus neutralization assay has a relatively fixed virus dose used in one-round infections. Importantly, in live-virus assays, there may be the cell-to-cell viral spread that is absent in pseudovirus neutralization assays. Cell-cell fusion is typically less sensitive to nAbs neutralization than cell-free virion infection. Thus, higher binding affinity to spike may not always be directly translatable into higher potency in live virus neutralization assays.

Importantly, F6-ab8-Fc also reduced lung viral titers in mice infected with the Beta variant and protected against mortality when administered prophylactically. In addition to viral neutralization, Fc-effector functions are important for Ab protection *in vivo* (Ullah et al., 2021; Winkler et al., 2021). Our ELISA data showed that F6-Ab8-Fc binds to human CD64, CD32, and CD16A similarly as compared to human IgG1-Fc. F6-ab8-Fc shows high binding to CD64 and moderate binding to CD32A and CD16A (Figure S5E).

In summary, we have identified a broadly neutralizing antibody domain (V_H_ F6) with a unique paratope and epitope, and which neutralized all SARS-CoV-2 variants tested. The F6 epitope may be targeted to elicit broadly neutralizing Abs and vaccines against circulating SARS-CoV-2 variants. The biparatopic bispecific Ab, F6-ab8-Fc, with its broad neutralization activity and *in vivo* activity presents a new Ab therapeutics against current SARS-CoV-2 VOCs.

### Limitations of this study

While we identify and characterize a potent biparatopic molecule, our study has limitations. The strong binding to CD64 could suggest that this molecule has cell-mediated phagocytosis (ADCP) activity, and binding to CD16A may help to mobilize ADCC killing infected cells. However, Fc receptor binding may also have the potential to contribute to the immunopathology of SARS-CoV-2 (via antibody-dependent enhancement). The detailed role of Fc effector function for F6-ab8-Fc in the protection of mice from lethal SARS-CoV-2 challenge needs to be further investigated by future studies. Moreover, it is possible that future VOCs may evade F6-ab8-Fc, and thus screening and testing of this molecule should continue as new VOCs emerge. On ab8-resistant variant Omicron BA.1, the higher neutralization potency of F6-F6-Fc than F6-ab8-Fc highlights the more important role of avidity compared to biparatopicity. It remains to be seen whether F6-F6-Fc outperforms F6-ab8-Fc (thus monoparatopic avidity outweighs biparatopicity) on other SARS-CoV-2 VOCs such as Alpha and Delta.

## STAR★Methods

### Key resources table

**Table:** 

REAGENT or RESOURCE	SOURCE	IDENTIFIER
Phage display library		
VH phage library	(Li et al., 2020)	N/A

**Antibodies**		

VH F6	This paper	N/A
VH ab8	(Li et al., 2020)	N/A
F6-F6	This paper	N/A
F6-ab8	This paper	N/A
F6-Fc	This paper	N/A
VH-Fc ab8	(Li et al., 2020)	N/A
F6-ab8-Fc	This paper	N/A
Anti-FLAG-HRP	Sigma-Aldrich	Cat# A8592-1MG; RRID: AB_439702
IgG1 m336	(Ying et al., 2014)	N/A
anti-Human Fc-HRP	Sigma-Aldrich	Cat# A0170-1ML; RRID: AB_257868

**Bacterial and virus strains**		

TG1	Lucigen	Cat# 60502-1
DH5α	Lucigen	Cat# 60602-1
vaccinia virus VTF7.3	NIH	Cat# 356
vaccinia virus VCB21R	NIH	Cat# 3365
SARS-CoV-2 Pseudovirus WT (+D614G)	This paper	N/A
SARS-CoV-2 Pseudovirus Alpha	This paper	N/A
SARS-CoV-2 Pseudovirus Beta	This paper	N/A
SARS-CoV-2 Pseudovirus Gamma	This paper	N/A
SARS-CoV-2 Pseudovirus Delta	This paper	N/A
SARS-CoV-2 Pseudovirus Omicron BA.1	This paper	N/A
SARS-CoV-2 Pseudovirus Omicron BA.2	This paper	N/A
SARS-CoV-2 variant WT	BEI Resources	Cat# NR-52281
SARS-CoV-2 variant Alpha	BEI Resources	Cat# NR-54011
SARS-CoV-2 variant Beta	BEI Resources	Cat# NR-54008
SARS-CoV-2 variant Delta	BEI Resources	Cat# NR-55611,
SARS-CoV-2 variant Omicron BA.1	BEI Resources	Cat# NR-56461
SARS-CoV-2 mouse-adapted 10 (MA10) Beta variant	(Martinez et al., 2021a)	N/A

**Chemicals, peptides, and recombinant proteins**		

SARS2 RBD WT	(Li et al., 2020)	N/A
SARS2 RBD Beta	Sino Biological	Cat# 40592-V08H85
SARS2 RBD Omicron BA.1	Sino Biological	Cat# 40592-V08H121
SARS2 RBD F342L	Sino Biological	Cat# 40592-V08H6
SARS2 RBD N354D	Sino Biological	Cat# 40592-V08H2
SARS2 RBD N354D/D364Y	Acrobiosystems	Cat# SPD-S52H3
SARS2 RBD V367F	Sino Biological	Cat# 40592-V08H1
SARS2 RBD R408I	Sino Biological	Cat# 40592-V08H10
SARS2 RBD Q414R	Sino Biological	Cat# 40592-V08H44
SARS2 RBD K417N	Sino Biological	Cat# 40592-V08H59
SARS2 RBD W436R	Sino Biological	Cat# 40592-V08H9
SARS2 RBD N439K	Sino Biological	Cat# 40592-V08H14
SARS2 RBD N440K	Sino Biological	Cat# 40592-V08H55
SARS2 RBD K444R	Sino Biological	Cat# 40592-V08H54
SARS2 RBD K444N	This paper	N/A
SARS2 RBD G446V	Sino Biological	Cat# 40592-V08H51
SARS2 RBD G446S	Sino Biological	Cat# 40592-V08H76
SARS2 RBD L452R	Sino Biological	Cat# 40592-V08H28
SARS2 RBD Y453F	Sino Biological	Cat# 40592-V08H80
SARS2 RBD K458R	Sino Biological	Cat# 40592-V08H7
SARS2 RBD A475V	Sino Biological	Cat# 40592-V08H50
SARS2 RBD S477N	Sino Biological	Cat# 40592-V08H46
SARS2 RBD T478I	Sino Biological	Cat# 40592-V08H30
SARS2 RBD P479S	Sino Biological	Cat# 40592-V08H57
SARS2 RBD V483A	Sino Biological	Cat# 40592-V08H5
SARS2 RBD E484K	Sino Biological	Cat# 40592-V08H84
SARS2 RBD E484Q	Sino Biological	Cat# 40592-V08H81
SARS2 RBD E484D	Sino Biological	Cat# 40592-V08H104
SARS2 RBD F486S	Sino Biological	Cat# 40592-V08H74
SARS2 RBD N487R	Sino Biological	Cat# 40592-V08H75
SARS2 RBD F490L	Sino Biological	Cat# 40592-V08H83
SARS2 RBD F490S	Sino Biological	Cat# 40592-V08H41
SARS2 RBD Q493R	This paper	N/A
SARS2 RBD Q493L	This paper	N/A
SARS2 RBD S494P	Sino Biological	Cat# 40592-V08H18
SARS2 RBD N501Y	Sino Biological	Cat# 40592-V08H82
SARS2 RBD K417N/E484K/N501Y	Sino Biological	Cat# 40592-V08H85-B
SARS2 S1 K417N, E484K, and N501Y	Sino Biological	Cat# 40591-V08H10
SARS2 S1 WT	Sino Biological	Cat# 40591-V08B1
SARS2 S1 Alpha	Sino Biological	Cat# 40591-V08H7
SARS2 S1 Beta	Sino Biological	Cat# 40591-V08H10-B
SARS2 S trimer Alpha	Sino Biological	Cat# 40589-V08H12
SARS2 S trimer Beta	Sino Biological	Cat# 40589-V08H13
SARS2 S trimer Gamma	Sino Biological	Cat# 40589-V08H23
SARS2 S trimer Kappa	Sino Biological	Cat# 40589-V08H11
SARS2 S trimer Delta	Sino Biological	Cat# 40589-V08H10
SARS2 S trimer Omicron BA.1	Acrobiosystems	Cat# SPN-C5224
SARS2 S trimer Omicron BA.2	Acrobiosystems	Cat# SPN-C5223
hACE2-mFc (mouse Fc)	Sino Biological	Cat# 10108-H05H
RBD-Fc	(Li et al., 2020)	N/A
Recombinant FcγRIA	Sino Biological	Cat# 10256-H08H
Recombinant FcγRIIA	Sino Biological	Cat# 10374-H08H
Recombinant FcγRIIIA	Sino Biological	Cat# 10389-H08H1

**Critical commercial assays**		

Blitz Protein A sensor	ForteBio	Cat# 18-5010
Blitz Streptavidin sensor	ForteBio	Cat# 18–5019
QuikChange II XL Kit	Agilent	Cat# 200521
β-galactosidase assay kit	G-Biosciences	Cat# 786-651
ONE-Glo™ EX Luciferase Assay System	Promega	Cat# E8110
Nano-Glo Assay System	Promega	Cat# N1110
Lenti-X™ GoStix™ Plus	TaKaRa	Cat# 631280
BirA biotin-protein ligase standard reaction kit	Avidity,	Cat# BirA500

**Deposited data**		

F6 antibody sequence	GENEBANK	ID: ON855352
F6/Beta spike CryoEM map	EMDB	EMD-27438 and EMD-27439
F6/Beta spike CryoEM structure	PDB	ID: 8DI5

**Experimental models: Cell lines**		

293T	ATCC	ATCC® CRL-3216
293T-S (WT)	(Li et al., 2020)	N/A
293T-hACE2	(Li et al., 2020)	N/A
Expi293F	ThermoFisher	Cat# A14527
Vero-E6	ATCC	ATCC® CRL-1586
HEK293T-ACE2-TMPRSS2 cells	BEI Resources	Cat# NR-55293

**Experimental models: Organisms/strains**		

BALB/c mice	Envigo	Cat# 047

**Recombinant DNA**		

Plasmid: pcDNA3.1-spike-D614G	This paper	N/A
Plasmid: pcDNA3.1-spike-Alpha	This paper	N/A
Plasmid: pcDNA3.1-spike-Beta	This paper	N/A
Plasmid: pcDNA3.1-spike-Gamma	This paper	N/A
Plasmid: pcDNA3.1-spike-Delta	This paper	N/A
Plasmid: pcDNA3.1-spike-Omicron BA.1	This paper	N/A
Plasmid: pcDNA3.1-spike-Omicron BA.2	This paper	N/A
Plasmid: pcDNA3.1-RBD-mutant K444N	This paper	N/A
Plasmid: pcDNA3.1-RBD-mutant Q493R	This paper	N/A
Plasmid: pcDNA3.1-RBD-mutant Q493L	This paper	N/A
Plasmid: pIW-Zeo-F6-F6-His	This paper	N/A
Plasmid: pIW-Zeo-F6-F6-Fc	This paper	N/A
Plasmid: pIW-Zeo-F6-ab8-His	This paper	N/A
Plasmid: pIW-Zeo-F6-ab8-Fc	This paper	N/A

**Software and algorithms**		

GraphPad Prism	GraphPad 9.0	https://www.graphpad.com/scientific-software/prism/
Snapgene	GSL Biotech LLC	https://www.snapgene.com/
PyMoL	Schrödinger	https://pymol.org/2/
FlowJ	FlowJo,V10, LLC	https://www.flowjo.com/solutions/flowjo/downloads
EPU automated acquisition	ThermoFisher Scientific	https://www.thermofisher.com/us/en/home/electron-microscopy/products/software-em-3d-vis/epu-software.html
UCSF Chimera v.1.15	(Pettersen et al., 2004)	https://www.cgl.ucsf.edu/chimera/
cryoSPARC v.3.2	(Punjani et al., 2017)	https://cryosparc.com/live
COOT v.0.9.3	(Emsley et al., 2010)	https://www2.mrc-lmb.cam.ac.uk/personal/pemsley/coot/binaries/release/
Phenix v.1.19	(Afonine et al., 2018)	https://phenix-online.org/download/
MolProbity	(Chen et al., 2010)	http://molprobity.biochem.duke.edu/
ChimeraX v.1.1.1	(Goddard et al., 2018)	https://www.cgl.ucsf.edu/chimerax/
NAMD (version 2.13)	(Phillips et al., 2005)	https://www.ks.uiuc.edu/Research/namd/
Modeller	(Fiser and Sali, 2003)	https://salilab.org/modeller/
PRODIGY	(Xue et al., 2016)	https://wenmr.science.uu.nl/prodigy/

### Resource availability

#### Lead contact

Further information and requests for resources and reagents should be directed to and will be fulfilled by the Lead Contact, Wei Li (LIWEI171@pitt.edu).

#### Materials availability

All requests for resources and reagents should be directed to and will be fulfilled by the Lead Contact author. This includes antibodies, viruses, plasmids and proteins. All reagents will be made available on request after completion of a Material Transfer Agreement.

### Experimental model and subject details

#### Cells and virus

Vero E6 (CRL-1586, American Type Culture Collection (ATCC) and 293T (ATCC) were cultured at 37°C in Dulbecco’s Modified Eagle medium (DMEM) supplemented with 10% fetal bovine serum (FBS), 10 mM HEPES pH 7.3, 1 mM sodium pyruvate, and 100 U/mL of penicillin–streptomycin. 293T was cultured in DMEM medium. 293T-Spike and 293T-ACE2 were cultured in DMEM medium containing 100 μg/mL Zeocin. Expi293F was maintained in Expi293™ Expression Medium (ThermoFisher, Cat# A1435103). The SARS-CoV-2 spike pseudotyped HIV-1 backboned virus were packaged in 293T cells after transfecting pNL4-3.luc.RE and pcDNA3.1-spike plasmids (WT, Alpha, Beta, Gamma, Delta, Omicron BA.1 and Omicron B.2). The SARS-CoV-2 live virus variants (WT, Alpha, Beta, Delta, Omicron BA.1) ordered from BEI Resources and propagated VeroE6 cells. The mouse ACE2 adapted SAR-CoV-2 virus (Beta variants) gene recovered by the reverse genetics was produced in VeroE6 cells. All work with infectious SARS-CoV-2 was performed in Institutional Biosafety Committee approved BSL3 facilities using appropriate positive pressure air respirators and protective equipment.

#### Recombinant proteins

The recombinant proteins RBD mutants (K444N, Q439R and Q439L) and RBD-Fc were subcloned into pcDNA3.1 or pIW-Zeo expression plasmids, and expressed in Expi293F cells. Proteins with his tag were purified by Ni-NTA affinity chromatography and protein with Fc tag purified by protein A chromatography. Protein purity was estimated as >95% by SDS-PAGE and protein concentration was measured spectrophotometrically (NanoVue, GE Healthcare).

#### Monoclonal antibodies

V_H_ F6 antibody was identified by panning of the phage library. VH ab8 was previously identified by our lab. F6-F6, F6-Fc, F6-ab8-Fc, F6-F6-Fc were cloned into pIW-Zeo expression plasmids, and expressed in Expi293F cells. MERS-CoV-specific IgG1 m336 sequences cloned into the pDR12 plasmid and expressed in Expi293F cells. V_H_ ab8 and V_H_ F6 (in a phagemid pComb3x with a Flag tag) was expressed in HB2151 *E. coli*. Antibodies with his tag were purified by Ni-NTA affinity chromatography and antibodies with Fc tag purified by protein A chromatography.

#### Mouse experiments

For the mouse model, BALB/c mice purchased from Envigo (BALB/cAnNHsd, stock# 047, immunocompetent, 11–12 months of age, female) were used for all experiments. They are drug/test naïve and negative for pathogens. Animals were not involved in any previous studies. Animals were housed in groups of 5 animals per cage and fed standard chow diet. The study was carried out in accordance with the recommendations for care and use of animals by the Office of Laboratory Animal Welfare (OLAW), National Institutes of Health and the Institutional Animal Care. All mouse studies were performed at the University of North Carolina (Animal Welfare Assurance #A3410-01) using protocols (19-168) approved by the UNC Institutional Animal Care and Use Committee (IACUC) and all virus studies were performed in ABSL3 facilities at UNC. Virus inoculations were performed under anesthesia and all efforts were made to minimize animal suffering. For evaluating prophylactic efficacy of F6-ab8-Fc, mice were intraperitoneally treated (12 h before infection) with different doses of F6-ab8-Fc followed by intranasal challenge with 10^5^ PFU of mouse-adapted SARS-CoV-2 Beta variant. For evaluating prophylactic efficacy of F6-ab8-Fc, mice were intraperitoneally treated (12 h before infection) with 800 μg or 50 μg of F6-ab8-Fc followed by intranasal challenge with 10^5^ PFU of mouse-adapted SARS-CoV-2 Beta variant. For evaluating the therapeutic efficacy of F6-ab8-Fc, mice were intraperitoneal injection with 800 μg or 50 μg of F6-ab8-Fc 12 h following infection. Four days post infection, mice were sacrificed and perfused with 10 mL PBS. Then lung was harvested for viral titer as determined by the plaque assay.

### Method details

#### Antigen expression and phage panning

The SARS-CoV-2 RBD, S1 and S trimer mutants were ordered from Sino Biological (USA). The VH F6 and VH ab8 were expression in HB2151 bacteria cells as previously described (Chen et al., 2021; Sun et al., 2020). F6-F6, F6-Fc, F6-ab8-Fc, F6-F6-Fc, and RBD-Fc were expressed with Expi293 cells as previously described (Li et al., 2020; Sun et al., 2020). Expressed protein purity was estimated as >95% by SDS-PAGE (Invitrogen) and protein concentration was measured spectrophotometrically (NanoVue, GE Healthcare). The panning process was described in detail in our previous protocol (Chen et al., 2021).

#### ELISA

Ninety-six-well ELISA plates (Corning 3690) were coated with the RBD, S1 mutants or S trimer variants at a concentration of 5 μg /mL (diluted with 1xPBS) and incubated at 4°C overnight (50 μL per well). The next day, plates were blocked with 150 μL 5% milk (Bio-Rad) in DPBS solution at room temperature for 2 h. Primary antibodies were diluted with the same 5% milk blocking buffer and 1:10 or 1:3 serial dilution series were conducted, with 1 μM as the highest concentration. After 2 h of blocking, the primary antibodies were added (50 μL per well) and incubated at room temperature for 2 h. After 2 h incubation, the plates were washed 4 times with 0.05% Tween 1xPBS (PBST) solution using a plate washer (BioTek). Secondary antibodies (anti-Flag-HRP or anti-Human Fc-HRP) were prepared with the same 5% milk at a dilution of 1:1000. 50 μL of secondary antibody was added into each well and incubated at room temperature for 1 h. To test F6-ab8-Fc binding to human FcγRs, F6-ab8-Fc was coated on plates followed by addition of the recombinant human FcγR protein in gradient concentrations. After washing, the binding was detected by HRP conjugated anti-His tag Ab. For testing binding of VH F6 and F6-ab8-Fc to Omicron BA.1 and BA.2 RBD and spike proteins, the RBD or spike were coated, and binding were detected by using HRP anti-FLAG tag for VH F6 and the HRP anti-human Fc Ab for F6-ab8-Fc. After 1 h incubation, the plates were washed 5 times with PBST. Fifty μL of TMB substrate (Sigma) was added into each well, allowed 1-2 min to develop color, then stopped with 50 μL H2SO4 (1M, Sigma) and the plate scanned at 450 nm absorbance. The ELISA results were analyzed using GraphPad Prism 9.0.2.

#### BLItz

Antibody affinities were measured by biolayer interferometry BLItz (ForteBio, Menlo Park, CA). For VH F6 affinity determination, VH F6 was biotinylated with BirA biotin-protein ligase standard reaction kit (BirA500, Avidity, USA). Streptavidin biosensors (ForteBio: 18–5019) were used for biotinylated VH F6 immobilization. For F6-ab8-Fc affinity determination, Protein A biosensors (ForteBio: 18-5010) were used for immobilization. Dulbecco’s phosphate-buffered saline (DPBS) (pH = 7.4) was used for baseline and dissociation collection. The detection conditions used were: (I) baseline 30s; (II) loading 120 s; (III) baseline 30 s; (IV) association 120 s with a series of concentrations (1000 nM, 500 nM, 250 nM for VH F6; 500 nM, 250 nM, 125 nM for F6-ab8-Fc); (V) dissociation 240 s. The Ka and Kd rates were measured by BLItz software and KD was calculated for each antibody by the Kd /Ka ratio. For VH F6 - VH ab8 competition, Protein A biosensors (ForteBio: 18-5010) were used for RBD-Fc immobilization. The detection conditions used were (I) baseline 30s; (II) loading 120 s; (III) baseline 30 s; (IV) association 120 s with VH ab8; (V) association 120 s with VH F6.

#### Electron microscopy sample preparation and data collection

For cryo-EM, SARS-CoV-2 S trimer Beta mutant were deposited on grids at a final concentration of 2 mg/mL. Complexes were prepared by incubating S trimer Beta mutant with VH F6 at a molar ratio of 1:10. Grids were cleaned with H2/O2 gas mixture for 15 s in PELCO easiGlow glow discharge unit (Ted Pella) and 1.8 μL of protein suspension was applied to the surface of the grid. Using a Vitrobot Mark IV (Thermo Fisher Scientific), the sample was applied to either Quantifoil Holey Carbon R1.2/1.3 copper 300 mesh grids or UltrAuFoil Holey Gold 300 mesh grids at a chamber temperature of 10°C with a relative humidity level of 100%, and then vitrified in liquid ethane after blotting for 12 s with a blot force of −10. All cryo-EM grids were screened using a 200-kV Glacios (Thermo Fisher Scientific) TEM equipped with a Falcon4 direct electron detector and data were collection on a 300-kV Titan Krios G4 (Thermo Fisher Scientific) TEM equipped with a Falcon4 direct electron detector in electron event registration (EER) mode. Movies were collected at 155,000× magnification (physical pixel size 0.5 Å) over a defocus range of −3 μm to −0.5 μm with a total dose of 40 e – /Å2 using EPU automated acquisition software (Thermo Fisher).

#### Image processing

A detailed workflow for the data processing is summarized in Figure S2. All data processing was performed in cryoSPARC v.3.2 (Punjani et al., 2017). On-the-fly data pre-processing including patch mode motion correction (EER upsampling factor 1, EER number of fractions 40), patch mode CTF estimation, reference free particle picking, and particle extraction were carried out in cryoSPARC live. Next, particles were subjected to 2D classification (just for evaluation of the data quality) and 3 rounds of 3D heterogeneous classification. The global 3D refinement was performed with per particle CTF estimation and high-order aberration correction. Focused refinement was performed with a soft mask covering the down RBD and its bound VH F6. Resolutions of both global and local refinements were determined according to the gold standard FSC (Bell et al., 2016).

#### Model building and refinement

Initial models either from published coordinates (PDB code 7MJI) or from homology modeling (V_H_ F6) (Waterhouse et al., 2018) were docked into the focused refinement maps or global refinement maps using UCSF Chimera v.1.15 (Pettersen et al., 2004). Then, mutation and manual adjustment were performed with COOT v.0.9.3 (Emsley et al., 2010), followed by iterative rounds of refinement in COOT and Phenix v.1.19 (Afonine et al., 2018). Model validation was performed using MolProbity (Chen et al., 2010). Figures were prepared using UCSF Chimera, UCSF ChimeraX v.1.1.1 (Goddard et al., 2018), and PyMOL (v.2.2 Schrodinger, LLC).

#### Molecular dynamics simulations of SARS-CoV-2 omicron RBD complexed with F6, and evaluation of binding energies

We constructed a structural model for the Omicron RBD complexed with F6 using the cryo-EM structure of F6/Beta RBD complex as template, and constructed the system for molecular dynamics simulations of this complex using the CHARMM-GUI Solution Builder module (Jo et al., 2008). The resolved N-linked glycans and disulphides were included in the model, along with explicit water molecules to cover a distance 10 Å away from protein edges. Sodium and chloride ions corresponding to 0.15 M NaCl were included. This resulted in a simulation box of 94×94×94 Å3. CHARMM36 force field with CMAP corrections was used for the protein, water, and glycan molecules (Guvench et al., 2011; Huang et al., 2017). All MD simulations were performed using NAMD (version 2.13) (Phillips et al., 2005) with the protocol adopted from earlier work. Simulations were performed in triplicates with 100 ns each for the Omicron RBDs complexed with F6. Binding free energies ΔGbinding were evaluated using PRODIGY (Xue et al., 2016), and binding dissociation constants, KD, using KD = exp(ΔGbinding/RT) x 109 (in nM) with RT = 0.6 kcal/mol at T = 300K. ΔGbinding histograms were generated based on 800 snapshots evenly collected during the MD simulation time interval 20 < t ≤ 100 ns for each run. The F6-ab8-Fc structure was modeled by using Modeller (Fiser and Sali, 2003) based on homology modeling using multiple templates. VH F6 and ab8 moieties were based on the experimental resolved cryoEM structure (Zhu et al., 2021), while the Fc fragment was modeled based the structure of full-length antibody (Scapin et al., 2015). The distance of the two VH F6 moiety can be varied (between 7-16 nm) by loop refinement of the linker conformations using Modeller.

#### Pseudovirus neutralization assay

SARS-CoV-2 spike Wuhan-Hu-1 (+D614G), Alpha, Beta, Gamma, Delta, and Omicron protein genes were synthesized and inserted into pcDNA3.1 (GeneArt Gene Synthesis, Thermo Fisher Scientific). HEK293T cells (ATCC, cat#CRL-3216) were used to produce pseudotyped retroviral particles as described previously (Crawford et al., 2020). 60 h post transfection, pseudoviruses were harvested and filtered with a 0.45 μm PES filter. HEK293T-ACE2-TMPRSS2 cells (BEI Resources cat# NR-55293) were seeded in 384-well plates at 20 000 cells for neutralization assays. 24 h later, normalized amounts of pseudovirus preparations (Lenti-X™ GoStix™ Plus) were incubated with dilutions of the indicated antibodies or media alone for 1 h at 37°C prior to addition to cells and incubation for 48 h. Cells were lysed and luciferase activity assessed using the ONE-Glo™ EX Luciferase Assay System (Promega) according to the manufacturer’s specifications. Detection of relative luciferase units (RLUs) was measured using a Varioskan Lux plate reader (Thermo Fisher).

#### Authentic SARS-CoV-2 plaque reduction neutralization assay

Neutralization assays were performed using Vero E6 cells (ATCC CRL-1586). One day before the assay, the Vero E6 cells (3 × 10^5^ cells) were seeded in 24-well tissue culture plates per well. Antibodies (VH F6 and F6-ab8-Fc) were serially diluted by two-fold with a starting concentration ranging from 4 μg/mL to 40 μg/mL (depending on the antibody being tested) and mixed with equal volume of 30-50 plaque forming units (pfu) of SARS-CoV-2. The following SARS-CoV-2 variants were used: isolate USA-WA1/2020 (NR-52281, BEI Resources); isolate hCoV-19/South Africa/KRISP-EC-K005321/2020 (NR-54008, BEI Resources); Alpha isolate USA/CA_CDC_5574/2020 (NR-54011, BEI Resources); Delta isolate hCoV-19/USA/PHC658/2021 (NR-55611, BEI Resources); Omicron BA.1 isolate hCoV-19/USA/MD-HP20874/2021 (NR-56461, BEI Resources). The antibody-virus mixture was then incubated at 37°C in a 5% CO2 incubator for 1 h before adding to the Vero E6 cell seeded monolayers. The experiments were performed in duplicate. Following 1 h incubation at 37°C, an overlay media containing 1% agarose (2x Minimal Essential Medium, 7.5% bovine albumin serum, 10 mM HEPES, 100 μg/mL penicillin G and 100 U/mL streptomycin) was added into the monolayers. The plates were then incubated for 48–72 h and then cells were fixed with formaldehyde for 2 h. Following fixation, agar plugs were removed, and cells were stained with crystal violet. To precisely titrate the input virus, a viral back-titration was performed using culture medium as a replacement for the antibodies. To estimate the neutralizing capability of each antibody, IC50 was calculated by non-linear regression using the sigmoidal dose response equation in GraphPad Prism 9. All assays were performed in the University of Pittsburgh Regional Biocontainment Laboratory BSL-3 facility.

#### Cell-cell fusion inhibition assay

A β-gal reporter gene based quantitative cell fusion assay (Liu et al., 2020b) was used to test the cell-cell fusion inhibitory activity of F6-ab8-Fc. Briefly, 293T-S (WT) cells were infected with vaccinia virus expressing T7 polymerase (vTF7-3, obtained from NIH), while 293T-ACE2 cells were infected with vaccinia virus (vCB21R Lac-Z) encoding the T7 promotor-controlled β-galactosidase. 293T-S cells were pre-mixed with 1 μM Abs at 37°C for 1h followed by incubation with 293T-ACE2 cells at a 1:1 ratio for 3h at 37°C. Then cells were then lysed, and the β-gal activity was measured using β-galactosidase assay kit (substrate CPRG, G-Biosciences, St. Louis, MO) following the manufacturer’s protocols. The incubation of 293T-S with 293T-ACE2 cells without additions of Abs, and incubation of 293T-S with 293T (without expressing ACE2) were set as positive and negative controls, respectively.

#### Evaluation of F6-ab8-Fc prophylactic and therapeutic efficacy with SARS-CoV-2 mouse models

Eleven to twelve-month old female immunocompetent BALB/c mice (Envigo, stock# 047) were used for SARS-CoV-2 *in vivo* Prophylactic and Therapeutic experiments as described previously (Martinez et al., 2021a, 2021b) Each group contains five mice and five mice per cage (contain one mouse from each group) and fed standard chow diet. To evaluate the prophylactic efficacy of F6-ab8-Fc, mice were intraperitoneal (i.p.) injection with 800 μg or 50 μg of F6-ab8-Fc 12 h prior virus infection. Mice were infected intranasally with 10^5^ plaque-forming units (PFU) of mouse-adapted SARS-CoV-2 B.1.351 MA10. For evaluating the therapeutic efficacy of F6-ab8-Fc, mice were intraperitoneal injection with 800 μg of or 50 μg of F6-ab8-Fc12 h following infection. 4 days after virus infection, mice were sacrificed, and lungs were harvested for viral titer by plaque assays. The caudal lobe of the right lung was homogenized in PBS. The homogenate was 10-fold serial-diluted and inoculated with confluent monolayers of Vero E6 cells at 37°C, 5% CO2 for 1 h. After incubation, 1 mL of a viscous overlay (1:1 2X DMEM and 1.2% methylcellulose) is added into each well. Plates are incubated for 4 days at 37°C, 5% CO2. Then, the plates are fixation, staining, washing and dried. Plaques of each plate are counted to determined virus titer. The study was carried out in accordance with the recommendations for care and use of animals by the Office of Laboratory Animal Welfare (OLAW), National Institutes of Health and the Institutional Animal Care. All mouse studies were performed at the University of North Carolina (Animal Welfare Assurance #A3410-01) using protocols (19-168) approved by the UNC Institutional Animal Care and Use Committee (IACUC) and all mouse studies were performed in a BSL3 facility at UNC.

### Quantification and statistical analysis

For ELISA, all the experiments were performed in duplicate and error bars denote ± SD, n = 2. For pseudovirus neutralization, all experiments were repeated at least twice in triplicate and error bars denote mean ± 1 SD, n = 3. For live virus neutralization, all experiments were repeated at least twice in triplicate and error bars denote mean ± 1 SD, n = 3. For the comparisons of F6-ab8-Fc and V_H_ F6 mediated inhibition of cell-to-cell fusion in the β-gal reporter assay, experiments were performed in triplicate. The paired Student t test was used to evaluate statistical differences. ∗p < 0.05, ∗∗p < 0.01. For the mouse model, the statistical significance of difference between F6-ab8-Fc treated and control mice lung virus titers was determined by the two-tailed, unpaired, student *t* test calculated using GraphPad Prism 9.0. A p value < 0.05 was considered significant. ns: p > 0.05, ∗p < 0.05, ∗∗p < 0.01, ∗∗∗p < 0.001.

## Data Availability

• Antibody nucleotide sequence has been deposited to GenBank. Accession number is listed in the key resources table. The F6/Beta spike Cryo-EM map has been uploaded to EMDB. Accession ID are listed in the key resources table. The F6/Beta spike Cryo-EM structure has been uploaded to PDB. Accession ID is listed in the key resources table. The antibody is only allowed for non-commercial use. • This paper does not report original code. • Any additional information required to reanalyze the data reported in this paper is available from the lead contact upon request.

## References

[bib1] Afonine P.V., Poon B.K., Read R.J., Sobolev O.V., Terwilliger T.C., Urzhumtsev A., Adams P.D. (2018). Real-space refinement in PHENIX for cryo-EM and crystallography. Acta Crystallogr. D Struct. Biol..

[bib2] Andreano E., Piccini G., Licastro D., Casalino L., Johnson N.V., Paciello I., Dal Monego S., Pantano E., Manganaro N., Manenti A. (2021). SARS-CoV-2 escape from a highly neutralizing COVID-19 convalescent plasma. Proc. Natl. Acad. Sci. USA.

[bib3] Barnes C.O., Jette C.A., Abernathy M.E., Dam K.-M.A., Esswein S.R., Gristick H.B., Malyutin A.G., Sharaf N.G., Huey-Tubman K.E., Lee Y.E. (2020). SARS-CoV-2 neutralizing antibody structures inform therapeutic strategies. Nature.

[bib4] Baum A., Fulton B.O., Wloga E., Copin R., Pascal K.E., Russo V., Giordano S., Lanza K., Negron N., Ni M. (2020). Antibody cocktail to SARS-CoV-2 spike protein prevents rapid mutational escape seen with individual antibodies. Science.

[bib5] Bell J.M., Chen M., Baldwin P.R., Ludtke S.J. (2016). High resolution single particle refinement in EMAN2.1. Methods (San Diego, Calif).

[bib6] Bracken C.J., Lim S.A., Solomon P., Rettko N.J., Nguyen D.P., Zha B.S., Schaefer K., Byrnes J.R., Zhou J., Lui I. (2021). Bi-paratopic and multivalent VH domains block ACE2 binding and neutralize SARS-CoV-2. Nat. Chem. Biol..

[bib7] Callaway E. (2021). Heavily mutated Omicron variant puts scientists on alert. Nature.

[bib8] Cameroni E., Bowen J.E., Rosen L.E., Saliba C., Zepeda S.K., Culap K., Pinto D., VanBlargan L.A., De Marco A., di Iulio J. (2022). Broadly neutralizing antibodies overcome SARS-CoV-2 Omicron antigenic shift. Nature.

[bib9] Cao L., Goreshnik I., Coventry B., Case J.B., Miller L., Kozodoy L., Chen R.E., Carter L., Walls A.C., Park Y.J. (2020). De novo design of picomolar SARS-CoV-2 miniprotein inhibitors. Science.

[bib10] Cao Y., Su B., Guo X., Sun W., Deng Y., Bao L., Zhu Q., Zhang X., Zheng Y., Geng C. (2020). Potent neutralizing antibodies against SARS-CoV-2 identified by high-throughput single-cell sequencing of convalescent patients' B cells. Cell.

[bib11] Cao Y., Yisimayi A., Jian F., Song W., Xiao T., Wang L., Du S., Wang J., Li Q., Chen X. (2022). BA.2.12.1, BA.4 and BA.5 escape antibodies elicited by Omicron infection. Nature.

[bib12] Chan K.K., Dorosky D., Sharma P., Abbasi S.A., Dye J.M., Kranz D.M., Herbert A.S., Procko E. (2020). Engineering human ACE2 to optimize binding to the spike protein of SARS coronavirus 2. Science.

[bib13] Chen C., Sun Z., Liu X., Li W., Dimitrov D.S. (2021). Protocol for constructing large size human antibody heavy chain variable domain (VH) library and selection of SARS-CoV-2 neutralizing antibody domains. STAR Protoc..

[bib14] Chen V.B., Arendall W.B., Headd J.J., Keedy D.A., Immormino R.M., Kapral G.J., Murray L.W., Richardson J.S., Richardson D.C. (2010). MolProbity: all-atom structure validation for macromolecular crystallography. Acta Crystallogr. D Biol. Crystallogr..

[bib15] Cho H., Gonzales-Wartz K.K., Huang D., Yuan M., Peterson M., Liang J., Beutler N., Torres J.L., Cong Y., Postnikova E. (2021). Bispecific antibodies targeting distinct regions of the spike protein potently neutralize SARS-CoV-2 variants of concern. Sci. Transl. Med..

[bib16] Collier D.A., De Marco A., Ferreira I.A.T.M., Meng B., Datir R.P., Walls A.C., Kemp S.A., Bassi J., Pinto D., Silacci-Fregni C. (2021). Sensitivity of SARS-CoV-2 B.1.1.7 to mRNA vaccine-elicited antibodies. Nature.

[bib17] Crawford K.H.D., Eguia R., Dingens A.S., Loes A.N., Malone K.D., Wolf C.R., Chu H.Y., Tortorici M.A., Veesler D., Murphy M. (2020). Protocol and reagents for pseudotyping lentiviral particles with SARS-CoV-2 spike protein for neutralization assays. Viruses.

[bib18] Cui J., Li F., Shi Z.L. (2019). Origin and evolution of pathogenic coronaviruses. Nat. Rev. Microbiol..

[bib19] De Gasparo R., Pedotti M., Simonelli L., Nickl P., Muecksch F., Cassaniti I., Percivalle E., Lorenzi J.C.C., Mazzola F., Magrì D. (2021). Bispecific IgG neutralizes SARS-CoV-2 variants and prevents escape in mice. Nature.

[bib20] Dong E., Du H., Gardner L. (2020). An interactive web-based dashboard to track COVID-19 in real time. Lancet Infect. Dis..

[bib21] Dong J., Zost S.J., Greaney A.J., Starr T.N., Dingens A.S., Chen E.C., Chen R.E., Case J.B., Sutton R.E., Gilchuk P. (2021). Genetic and structural basis for SARS-CoV-2 variant neutralization by a two-antibody cocktail. Nat. Microbiol..

[bib22] Emsley P., Lohkamp B., Scott W.G., Cowtan K. (2010). Features and development of coot. Acta Crystallogr. D Biol. Crystallogr..

[bib23] Fiser A., Sali A. (2003). Modeller: generation and refinement of homology-based protein structure models. Methods Enzymol..

[bib24] Geers D., Shamier M.C., Bogers S., den Hartog G., Gommers L., Nieuwkoop N.N., Schmitz K.S., Rijsbergen L.C., van Osch J.A.T., Dijkhuizen E. (2021). SARS-CoV-2 variants of concern partially escape humoral but not T-cell responses in COVID-19 convalescent donors and vaccinees. Sci. Immunol..

[bib25] Glasgow A., Glasgow J., Limonta D., Solomon P., Lui I., Zhang Y., Nix M.A., Rettko N.J., Zha S., Yamin R. (2020). Engineered ACE2 receptor traps potently neutralize SARS-CoV-2. Proc. Natl. Acad. Sci. USA.

[bib26] Goddard T.D., Huang C.C., Meng E.C., Pettersen E.F., Couch G.S., Morris J.H., Ferrin T.E. (2018). UCSF ChimeraX: meeting modern challenges in visualization and analysis. Protein Sci..

[bib27] Grabowski F., Kochańczyk M., Lipniacki T. (2022). The spread of SARS-CoV-2 variant omicron with a doubling time of 2.0–3.3 Days can Be explained by immune evasion. Viruses.

[bib28] Greaney A.J., Starr T.N., Barnes C.O., Weisblum Y., Schmidt F., Caskey M., Gaebler C., Cho A., Agudelo M., Finkin S. (2021). Mapping mutations to the SARS-CoV-2 RBD that escape binding by different classes of antibodies. Nat. Commun..

[bib29] Grein J., Ohmagari N., Shin D., Diaz G., Asperges E., Castagna A., Feldt T., Green G., Green M.L., Lescure F.X. (2020). Compassionate use of remdesivir for patients with severe covid-19. N. Engl. J. Med..

[bib30] Guvench O., Mallajosyula S.S., Raman E.P., Hatcher E., Vanommeslaeghe K., Foster T.J., Jamison F.W., MacKerell A.D. (2011). CHARMM additive all-atom force field for carbohydrate derivatives and its utility in polysaccharide and carbohydrate–protein modeling. J. Chem. Theor. Comput..

[bib31] Hansen J., Baum A., Pascal K.E., Russo V., Giordano S., Wloga E., Fulton B.O., Yan Y., Koon K., Patel K. (2020). Studies in humanized mice and convalescent humans yield a SARS-CoV-2 antibody cocktail. Science.

[bib32] Hastie K.M., Li H., Bedinger D., Schendel S.L., Dennison S.M., Li K., Rayaprolu V., Yu X., Mann C., Zandonatti M. (2021). Defining variant-resistant epitopes targeted by SARS-CoV-2 antibodies: a global consortium study. Science.

[bib33] Henderson R., Edwards R.J., Mansouri K., Janowska K., Stalls V., Gobeil S.M.C., Kopp M., Li D., Parks R., Hsu A.L. (2020). Controlling the SARS-CoV-2 spike glycoprotein conformation. Nat. Struct. Mol. Biol..

[bib34] Ho D., Wang P., Liu L., Iketani S., Luo Y., Guo Y., Wang M., Yu J., Zhang B., Kwong P. (2021). Increased resistance of SARS-CoV-2 variants B.1.351 and B.1.1.7 to antibody neutralization. Nature.

[bib35] Huang J., Rauscher S., Nawrocki G., Ran T., Feig M., de Groot B.L., Grubmüller H., MacKerell A.D. (2017). CHARMM36m: an improved force field for folded and intrinsically disordered proteins. Nat. Methods.

[bib36] Jiang W., Wang J., Jiao S., Gu C., Xu W., Chen B., Wang R., Chen H., Xie Y., Wang A. (2021). Characterization of MW06, a human monoclonal antibody with cross-neutralization activity against both SARS-CoV-2 and SARS-CoV. MAbs.

[bib37] Jo S., Kim T., Iyer V.G., Im W. (2008). CHARMM-GUI: a web-based graphical user interface for CHARMM. J. Comput. Chem..

[bib38] Kim C., Ryu D.K., Lee J., Kim Y.I., Seo J.M., Kim Y.G., Jeong J.H., Kim M., Kim J.I., Kim P. (2021). A therapeutic neutralizing antibody targeting receptor binding domain of SARS-CoV-2 spike protein. Nat. Commun..

[bib39] Klassen S.A., Senefeld J.W., Johnson P.W., Carter R.E., Wiggins C.C., Shoham S., Grossman B.J., Henderson J.P., Musser J., Salazar E. (2021). The effect of convalescent plasma therapy on mortality among patients with COVID-19: systematic review and meta-analysis. Mayo Clin. Proc..

[bib40] Kuba K., Imai Y., Rao S., Gao H., Guo F., Guan B., Huan Y., Yang P., Zhang Y., Deng W. (2005). A crucial role of angiotensin converting enzyme 2 (ACE2) in SARS coronavirus-induced lung injury. Nat. Med..

[bib41] Kumar S., Karuppanan K., Subramaniam G. (2022). Omicron (BA.1) and sub-variants (BA.1.1, BA.2, and BA.3) of SARS-CoV-2 spike infectivity and pathogenicity: a comparative sequence and structural-based computational assessment. J. Med. Virol..

[bib42] Lazarevic I., Pravica V., Miljanovic D., Cupic M. (2021). Immune evasion of SARS-CoV-2 emerging variants: what have we learnt so far?. Viruses.

[bib43] Li W., Schäfer A., Kulkarni S.S., Liu X., Martinez D.R., Chen C., Sun Z., Leist S.R., Drelich A., Zhang L. (2020). High potency of a bivalent human VH domain in SARS-CoV-2 animal models. Cell.

[bib44] Liu L., Wang P., Nair M.S., Yu J., Rapp M., Wang Q., Luo Y., Chan J.F.W., Sahi V., Figueroa A. (2020). Potent neutralizing antibodies against multiple epitopes on SARS-CoV-2 spike. Nature.

[bib45] Liu X., Drelich A., Li W., Chen C., Sun Z., Shi M., Adams C., Mellors J.W., Tseng C.T., Dimitrov D.S. (2020). Enhanced elicitation of potent neutralizing antibodies by the SARS-CoV-2 spike receptor binding domain Fc fusion protein in mice. Vaccine.

[bib46] Lv Z., Deng Y.Q., Ye Q., Cao L., Sun C.Y., Fan C., Huang W., Sun S., Sun Y., Zhu L. (2020). Structural basis for neutralization of SARS-CoV-2 and SARS-CoV by a potent therapeutic antibody. Science.

[bib47] Mannar D., Saville J.W., Zhu X., Srivastava S.S., Berezuk A.M., Tuttle K.S., Marquez A.C., Sekirov I., Subramaniam S. (2022). SARS-CoV-2 Omicron variant: antibody evasion and cryo-EM structure of spike protein-ACE2 complex. Science.

[bib48] Martinez D.R., Schäfer A., Gobeil S., Li D., De la Cruz G., Parks R., Lu X., Barr M., Stalls V., Janowska K. (2022). A broadly cross-reactive antibody neutralizes and protects against sarbecovirus challenge in mice. Sci. Transl. Med..

[bib49] Martinez D.R., Schäfer A., Leist S.R., De la Cruz G., West A., Atochina-Vasserman E.N., Lindesmith L.C., Pardi N., Parks R., Barr M. (2021). Chimeric spike mRNA vaccines protect against Sarbecovirus challenge in mice. Science.

[bib50] Martinez D.R., Schäfer A., Leist S.R., Li D., Gully K., Yount B., Feng J.Y., Bunyan E., Porter D.P., Cihlar T. (2021). Prevention and therapy of SARS-CoV-2 and the B.1.351 variant in mice. Cell Rep..

[bib51] Miao X., Luo Y., Huang X., Lee S.M.Y., Yuan Z., Tang Y., Chen L., Wang C., Wu F., Xu Y. (2020). A novel biparatopic hybrid antibody-ACE2 fusion that blocks SARS-CoV-2 infection: implications for therapy. MAbs.

[bib52] Monteil V., Kwon H., Prado P., Hagelkrüys A., Wimmer R.A., Stahl M., Leopoldi A., Garreta E., Hurtado Del Pozo C., Prosper F. (2020). Inhibition of SARS-CoV-2 infections in engineered human tissues using clinical-grade soluble human ACE2. Cell.

[bib53] Noy-Porat T., Makdasi E., Alcalay R., Mechaly A., Levy Y., Bercovich-Kinori A., Zauberman A., Tamir H., Yahalom-Ronen Y., Israeli M. (2020). A panel of human neutralizing mAbs targeting SARS-CoV-2 spike at multiple epitopes. Nat. Commun..

[bib54] Park Y.J., De Marco A., Starr T.N., Liu Z., Pinto D., Walls A.C., Zatta F., Zepeda S.K., Bowen J.E., Sprouse K.R. (2022). Antibody-mediated broad sarbecovirus neutralization through ACE2 molecular mimicry. Science.

[bib55] Pettersen E.F., Goddard T.D., Huang C.C., Couch G.S., Greenblatt D.M., Meng E.C., Ferrin T.E. (2004). UCSF Chimera--a visualization system for exploratory research and analysis. J. Comput. Chem..

[bib56] Phillips J.C., Braun R., Wang W., Gumbart J., Tajkhorshid E., Villa E., Chipot C., Skeel R.D., Kalé L., Schulten K. (2005). Scalable molecular dynamics with NAMD. J. Comput. Chem..

[bib57] Pinto D., Park Y.J., Beltramello M., Walls A.C., Tortorici M.A., Bianchi S., Jaconi S., Culap K., Zatta F., De Marco A. (2020). Cross-neutralization of SARS-CoV-2 by a human monoclonal SARS-CoV antibody. Nature.

[bib58] Prévost J., Finzi A. (2021). The great escape? SARS-CoV-2 variants evading neutralizing responses. Cell Host Microbe.

[bib59] Punjani A., Rubinstein J.L., Fleet D.J., Brubaker M.A. (2017). cryoSPARC: algorithms for rapid unsupervised cryo-EM structure determination. Nat. Methods.

[bib60] Robbiani D.F., Gaebler C., Muecksch F., Lorenzi J.C.C., Wang Z., Cho A., Agudelo M., Barnes C.O., Gazumyan A., Finkin S. (2020). Convergent antibody responses to SARS-CoV-2 in convalescent individuals. Nature.

[bib61] Scapin G., Yang X., Prosise W.W., McCoy M., Reichert P., Johnston J.M., Kashi R.S., Strickland C. (2015). Structure of full-length human anti-PD1 therapeutic IgG4 antibody pembrolizumab. Nat. Struct. Mol. Biol..

[bib62] Schoof M., Faust B., Saunders R.A., Sangwan S., Rezelj V., Hoppe N., Boone M., Billesbølle C.B., Puchades C., Azumaya C.M. (2020). An ultrapotent synthetic nanobody neutralizes SARS-CoV-2 by stabilizing inactive Spike. Science.

[bib63] Stanfield R.L., Dooley H., Verdino P., Flajnik M.F., Wilson I.A. (2007). Maturation of shark single-domain (IgNAR) antibodies: evidence for induced-fit binding. J. Mol. Biol..

[bib64] Starr T.N., Greaney A.J., Addetia A., Hannon W.W., Choudhary M.C., Dingens A.S., Li J.Z., Bloom J.D. (2021). Prospective mapping of viral mutations that escape antibodies used to treat COVID-19. Science.

[bib65] Sun Z., Chen C., Li W., Martinez D.R., Drelich A., Baek D.S., Liu X., Mellors J.W., Tseng C.T., Baric R.S., Dimitrov D.S. (2020). Potent neutralization of SARS-CoV-2 by human antibody heavy-chain variable domains isolated from a large library with a new stable scaffold. MAbs.

[bib66] Ullah I., Prévost J., Ladinsky M.S., Stone H., Lu M., Anand S.P., Beaudoin-Bussières G., Symmes K., Benlarbi M., Ding S. (2021). Live imaging of SARS-CoV-2 infection in mice reveals that neutralizing antibodies require Fc function for optimal efficacy. Immunity.

[bib67] Van Egeren D., Novokhodko A., Stoddard M., Tran U., Zetter B., Rogers M., Pentelute B.L., Carlson J.M., Hixon M., Joseph-McCarthy D., Chakravarty A. (2021). Risk of rapid evolutionary escape from biomedical interventions targeting SARS-CoV-2 spike protein. PLoS One.

[bib68] Wang K., Jia Z., Bao L., Wang L., Cao L., Chi H., Hu Y., Li Q., Zhou Y., Jiang Y. (2022). Memory B cell repertoire from triple vaccinees against diverse SARS-CoV-2 variants. Nature.

[bib69] Wang P., Nair M.S., Liu L., Iketani S., Luo Y., Guo Y., Wang M., Yu J., Zhang B., Kwong P.D. (2021). Antibody resistance of SARS-CoV-2 variants B.1.351 and B.1.1.7. Nature.

[bib70] Waterhouse A., Bertoni M., Bienert S., Studer G., Tauriello G., Gumienny R., Heer F.T., de Beer T.A.P., Rempfer C., Bordoli L. (2018). SWISS-MODEL: homology modelling of protein structures and complexes. Nucleic Acids Res..

[bib71] Weisblum Y., Schmidt F., Zhang F., DaSilva J., Poston D., Lorenzi J.C., Muecksch F., Rutkowska M., Hoffmann H.-H., Michailidis E. (2020). Escape from neutralizing antibodies by SARS-CoV-2 spike protein variants. eLife.

[bib72] Wibmer C.K., Ayres F., Hermanus T., Madzivhandila M., Kgagudi P., Oosthuysen B., Lambson B.E., de Oliveira T., Vermeulen M., van der Berg K. (2021). SARS-CoV-2 501Y.V2 escapes neutralization by South African COVID-19 donor plasma. Nat. Med..

[bib73] Winkler E.S., Gilchuk P., Yu J., Bailey A.L., Chen R.E., Chong Z., Zost S.J., Jang H., Huang Y., Allen J.D. (2021). Human neutralizing antibodies against SARS-CoV-2 require intact Fc effector functions for optimal therapeutic protection. Cell.

[bib74] Xue L.C., Rodrigues J.P., Kastritis P.L., Bonvin A.M., Vangone A. (2016). PRODIGY: a web server for predicting the binding affinity of protein-protein complexes. Bioinformatics.

[bib75] Ying T., Du L., Ju T.W., Prabakaran P., Lau C.C.Y., Lu L., Liu Q., Wang L., Feng Y., Wang Y. (2014). Exceptionally potent neutralization of Middle East respiratory syndrome coronavirus by human monoclonal antibodies. J. Virol..

[bib76] Yuan M., Wu N.C., Zhu X., Lee C.C.D., So R.T.Y., Lv H., Mok C.K.P., Wilson I.A. (2020). A highly conserved cryptic epitope in the receptor binding domains of SARS-CoV-2 and SARS-CoV. Science.

[bib77] Zhou D., Dejnirattisai W., Supasa P., Liu C., Mentzer A.J., Ginn H.M., Zhao Y., Duyvesteyn H.M.E., Tuekprakhon A., Nutalai R. (2021). Evidence of escape of SARS-CoV-2 variant B.1.351 from natural and vaccine-induced sera. Cell.

[bib78] Zhou T., Wang L., Misasi J., Pegu A., Zhang Y., Harris D.R., Olia A.S., Talana C.A., Yang E.S., Chen M. (2022). Structural basis for potent antibody neutralization of SARS-CoV-2 variants including B.1.1.529. Science.

[bib79] Zhu N., Zhang D., Wang W., Li X., Yang B., Song J., Zhao X., Huang B., Shi W., Lu R. (2020). A novel coronavirus from patients with pneumonia in China, 2019. N. Engl. J. Med..

[bib80] Zhu X., Mannar D., Srivastava S.S., Berezuk A.M., Demers J.P., Saville J.W., Leopold K., Li W., Dimitrov D.S., Tuttle K.S. (2021). Cryo-electron microscopy structures of the N501Y SARS-CoV-2 spike protein in complex with ACE2 and 2 potent neutralizing antibodies. PLoS Biol..

