# Supplementary material for: Potent and broad neutralization of SARS-CoV-2 variants of concern (VOCs) including omicron sub-lineages BA.1 and BA.2 by biparatopic human VH domains
Source: iScience. 2022 Jul 20;25(8):104798. doi: 10.1016/j.isci.2022.104798 (PMC9296231; doi:10.1016/j.isci.2022.104798)
Supplement: Document S1. Table S1 and Figures S1–S6 [file mmc1.pdf]

iScience, Volume *25*

Supplemental information

Potent and broad neutralization of SARS-CoV-2 variants of concern (VOCs) including omicron sub-lineages BA.1 and BA.2 by biparatopic human VH domains

Chuan Chen, James W. Saville, Michelle M. Marti, Alexandra Schäfer, Mary Hongying Cheng, Dhiraj Mannar, Xing Zhu, Alison M. Berezuk, Anupam Banerjee, Michele D. Sobolewski, Andrew Kim, Benjamin R. Treat, Priscila Mayrelle Da Silva Castanha, Nathan Enick, Kevin D. McCormick, Xianglei Liu, Cynthia Adams, Margaret Grace Hines, Zehua Sun, Weizao Chen, Jana L. Jacobs, Simon M. Barratt-Boyes, John W. Mellors, Ralph S. Baric, Ivet Bahar, Dimiter S. Dimitrov, Sriram Subramaniam, David R. Martinez, and Wei Li

Table S1. Cryo-EM density and model processing and validation parameters. Related to the Figure 2 Cryo-EM Structure

	S(Beta) + VH F6	
	global refinement	focused refinement
	(EMD-27438)	(EMD-27439)
		(PDB ID 8DI5)
**Data collection**		
Microscope	Titan Krios G4	
Detector	Falcon4	
Voltage (kV)	300	
Nominal magnification	155,000	
Defocus range (μm)	Falcon4	
Physical pixel (Å)	0.5	
Electron dose (e^{-}/Å^2)	40	
Exposure rate (e^{-}/Å^2/sec)	24	
Format of movies	EER	
Number of raw frames	399	
Number of movies	8,756	
**Data processing**		
Number of fractions	40	
Number of extracted particles	730,592	
Number of particles for final map	57,496	
Symmetry imposed	C1	C1
Resolution (Å)	2.83	3.04
FSC threshold	0.143	0.143
**Refinement**		
Initial model used		7MJI
Map sharpening B-factor (Å^2)	36.0	75.7
Composition (#)		
Atoms		2,467
Residues		311
Ligands		NAG:1
B-factor (Å^2)		
Protein (min/max/mean)		73.77/146.59/98.29
Ligand (min/max/mean)		99.30/99.30/99.30
Bonds (RMSD)		
Length (Å) (# > 4σ)		0.006 (0)
Angles (°) (# > 4σ)		0.946 (1)
CC_mask		0.80
**Validation**		
Ramachandran plot		
Residues favored (%)		96.74
Residues disallowed (%)		0.00
Rotamer outliers (%)		0.38
Clash score		4.17
MolProbity score		1.40

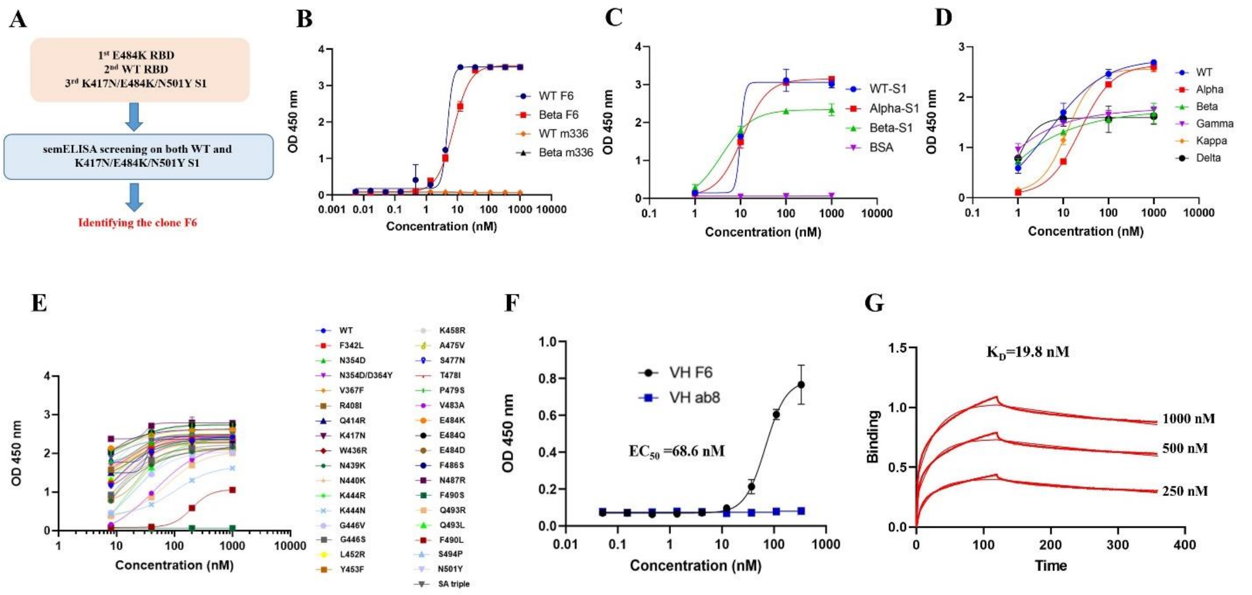

Figure S1. Identification of V_H F6 by a sequential phage panning strategy, which broadly binds to SARS-CoV-2 VOCs spike and RBD proteins. Related to the Figure 1: identification of the human antibody domain VH F6 with broad neutralization. A. Overview of phage panning strategy. Three rounds of panning were performed using the RBD E484K mutant for the first round, WT RBD for the second round and K417N/E484K/N501Y S1 protein for the third round of panning followed by supernatant expression monoclonal (sem)ELISA screening. **B**. ELISA results of V_H F6 binding to the WT and Beta RBD proteins. The MERS-CoV antibody IgG1 m336 was used as a negative control. **C**. V_H F6 binding to the SARS-CoV2 WT, Alpha and Beta S1 proteins. BSA was used as a negative control. **D**. V_H F6 binding to VOCs S trimer proteins measured by ELISA. **E**. V_H F6 binding to naturally occurring RBD mutants.. **F** and **G**. Measurement of V_H F6 binding to the Omicron BA.1 RBD by ELISA (**F**) and BLItz (**G**). All ELISA experiments were performed in duplicate and error bars denote ± SD, n=2.

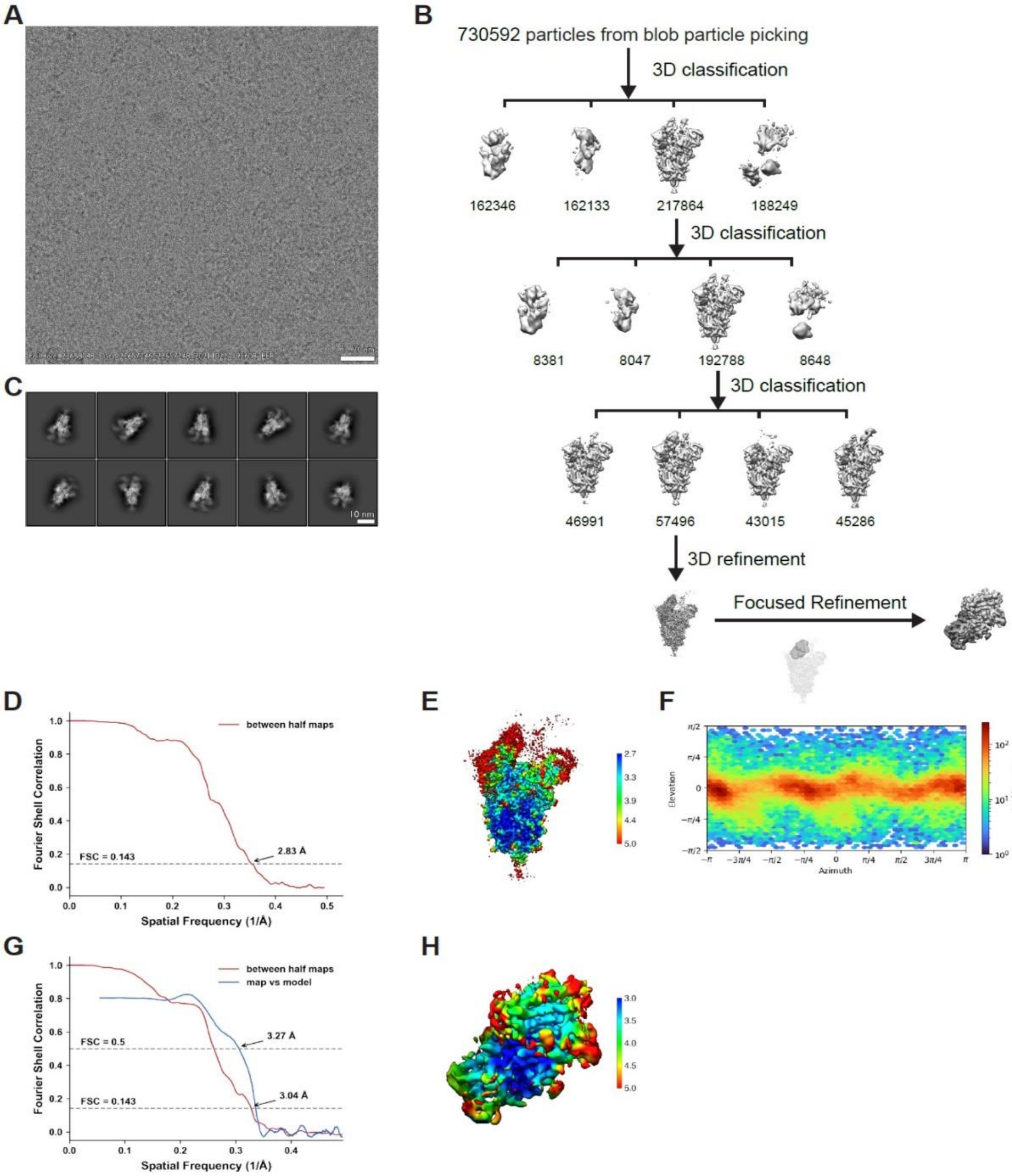

Figure S2. Cryo-EM data processing and validation for the VH F6 - Beta spike trimer complex. **Related to Figure 2. A**. Representative cryo-EM micrograph. **B**. Workflow of cryo-EM image processing. **C**. Representative 2D classes. (**D-F**) FSC curves (**D**), local resolution (**E**) and viewing direction distribution plot (**F**) of the global refinement. (**G-H**) FSC curves (**G**) and local resolution (**H**) of the focused refinement.

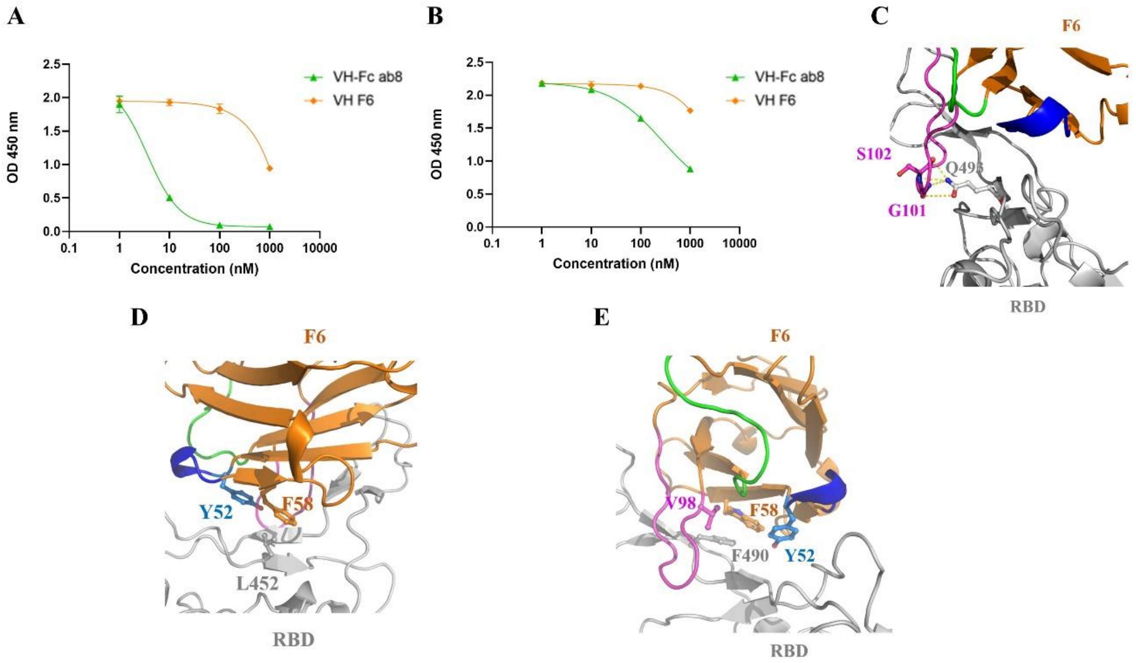

Figure S3. **ACE2 competition for V_H F6 and interfacial non-covalent interactions between F6 and RBD**. **Related to Figure 2.** Competition ELISA of V_H F6 and VH-Fc ab8 with hACE2 for binding to the RBD (**A**) and S trimer (**B**). ELISA experiments were performed in duplicate and error bars denote ± SD, n=2. **C-E**. V_H F6 - RBD interaction interface focusing on residues Q439 (**C**). L452 (**D**) and F490 (**E**). The RBD is shown as a gray cartoon, and V_H F6 as an orange cartoon with CDR1, CDR2 and CD3 highlighted with green, blue, and magenta colors respectively.

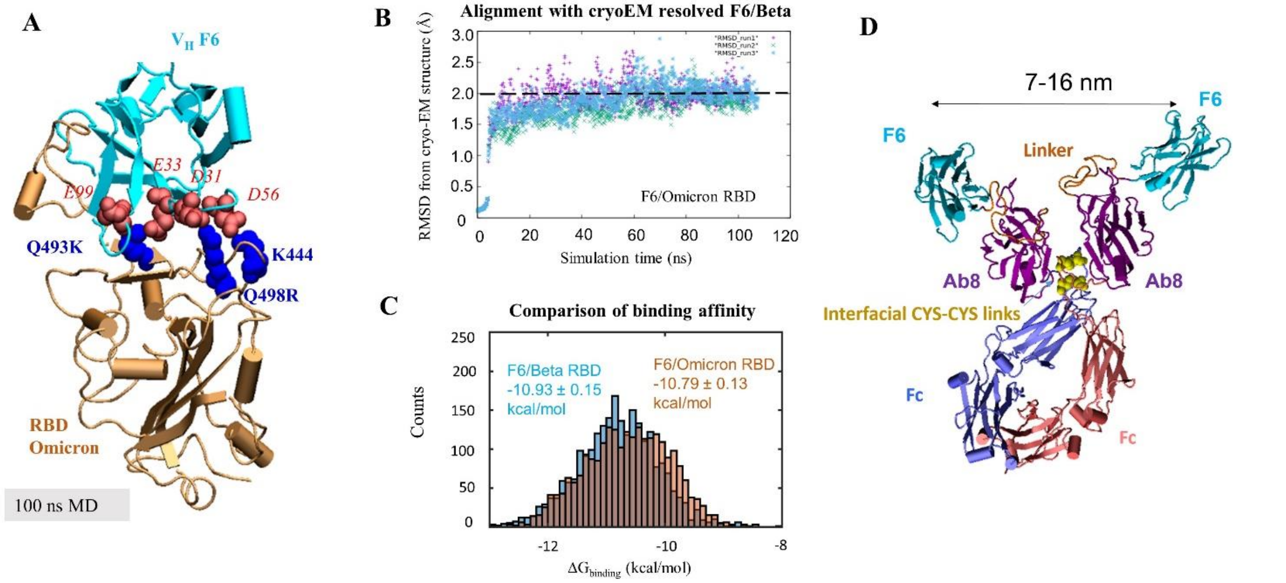

Figure S4. Modeling of the structure, equilibrium dynamics, and binding energetics of the complex between VH F6 and Omicron RBD, and modeling the structure of F6-ab8-Fc. Related to Star Methods "Molecular dynamics simulations of SARS-CoV-2 Omicron RBD complexed with F6, and evaluation of binding energies." (**A**) Structural model generated for the Omicron RBD-F6 complex. The diagram shows the conformation stabilized after 100 ns MD simulations, which retains features comparable to those of the cryoEM resolved F6-Beta RBD complex. (**B**) Stability of the F6/Omicron RBD complex near the conformation resolved for F6/Beta RBD complex. The root-mean square deviations (RMSDs) in the structural coordinates of F6/Omicron RBD with respect to the resolved F6/Beta RBD complex is shown as a function of simulation time. Purple, blue and green represent results taken from three different runs. The convergence to 2.0 ± 0.5 Å robustly reproduced in three different runs indicates the stability of the model that is closely similar to the cryo-EM structure resolved for F6/Beta RBD complex. (**C**) Histogram of the binding energies. Calculated based on three independent MD runs (800 evenly collected snapshots between 20 ns to 100 ns from each trajectory). The computed binding dissociation constants based on the average binding free energies are 12.2±3.1 and 15.5±3.3 nM,

for the F6/Beta RBD and F6/Omicron RBD, respectively. Standard deviations are estimated based on three different runs. (**D**). Structure modelling of F6-ab8-Fc. F6-ab8-Fc structure was modeled by using Modeller based on homology modeling. The distance of the two V_H F6 moiety can be varied (between 7-16 nm) with loop refinement of the linker conformations using Modeller.

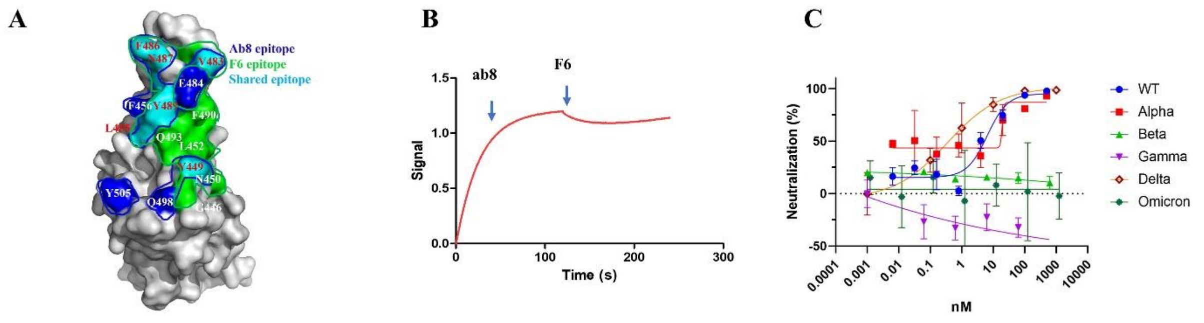

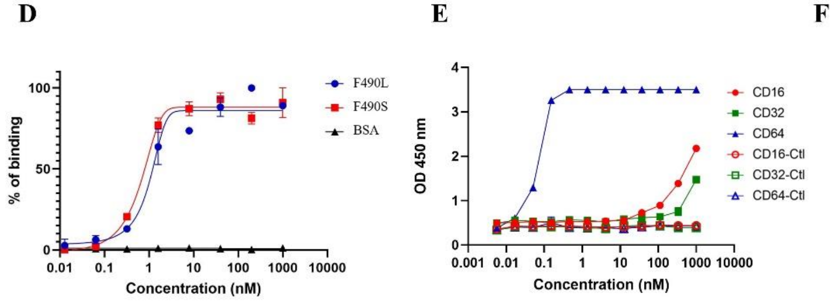

F

F6 constructs	Neutralization of Omicron BA.1 pseudovirus (IC_{50}, nM, mean ± 1 SD)
V_H F6	188.40[1]±116.74
F6-F6	143.70±61.16
F6-Fc	55.30±25.85
F6-ab8-Fc	21.50[2]±19.50
F6-F6-Fc	8.35±1.91

1, 2: These values are different from the ones in Fig.3D (268.9 nM for V_H F6, 10.86 nM for F6-ab8-Fc) due to batch-to-batch variations in the pseudovirus neutralization assays.

Figure S5. Rational design of the biparatopic F6-ab8-Fc, neutralization of SARS-CoV-2 VoCs pseudoviruses by VH-Fc ab8 and neutralization of Omicron BA.1 pseudovirus by different F6 constructs. **Related to Figure 3. A**. Comparison of the F6 epitope (green footprints) with the ab8 epitope (blue) on RBD surface. **B**. Competition of F6 with ab8 for binding to RBD as measured by BLItz. **C**. Neutralization of SARS-CoV2 VOCs pseudoviruses by ab8, which is escaped the by the Beta, Gamma, and Omicron variants. Experiments were performed in triplicate and error bars denote ± SD, n=3. **D**. ab8 is able to bind to the SARS-CoV-2 RBD mutants, F490L and F490S, which escape V_H F6 binding. ELISA experiments were performed in duplicate and error bars denote ± SD, n=2. **E**. Evaluation of binding F6-ab8-Fc to the recombinant human FcγR proteins by ELISA. Experiments were performed in duplicate and error bars denote ± SD, n=2. **F**. Side-by-side comparisons of neutralizations of Omicron BA.1 pseudovirus by different F6 fusion proteins with various molecular formats. Experiments were at least repeated twice and performed in triplicate and error bars denote ± SD, n=3.

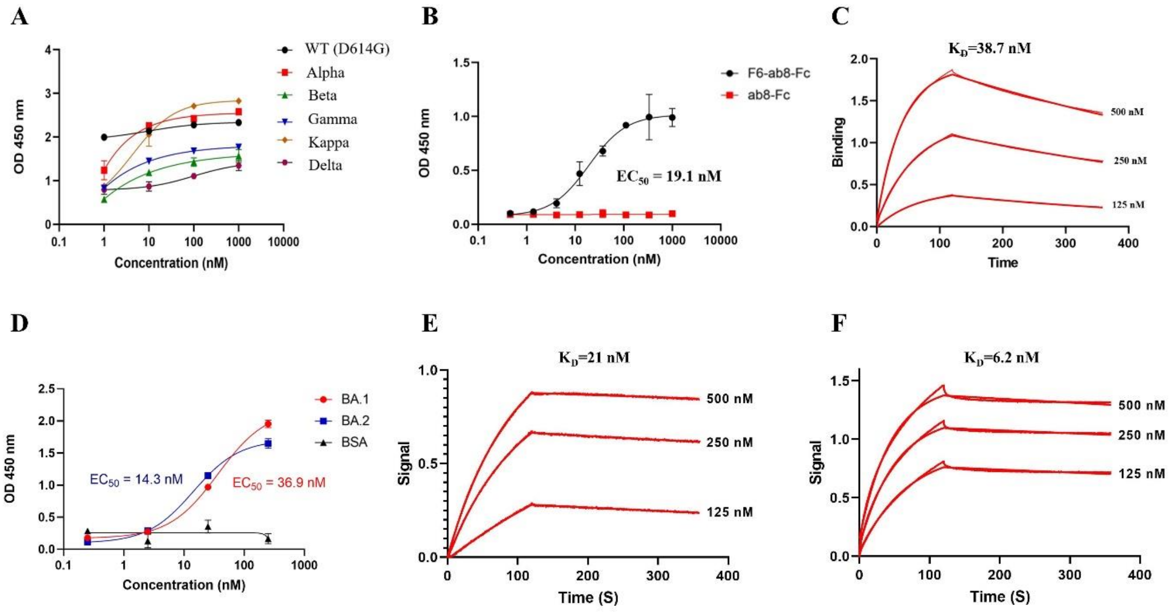

Figure S6. Binding of F6-ab8-Fc to SARS-CoV-2 VOCs trimeric Spike and RBD proteins tested by ELISA and BLItz. Related to Figure 3. A. Binding of F6-ab8-Fc to the recombinant SARS-CoV-2 VOCs trimeric spike proteins as measured by ELISA. Experiments were performed in duplicate and error bars denote ± SD, n=2. **B-C**. F6-ab8-Fc binding to the recombinant Omicron BA.1 RBD protein tested by ELISA. ELISA was performed in duplicate and error bars denote ± SD, n=2. (**B**) and BLItz (**C**). **D**. F6-ab8-Fc binding to the recombinant Omicron BA.1 and BA.2 spike proteins as tested by ELISA. Experiments were performed in duplicate and error bars denote ± SD, n=2. **E-F**. F6-ab8-Fc binding to the recombinant Omicron BA.1 and BA.2 spike proteins as tested by BLItz.
